# Melatonin activates the ZjERF34-ZjMYB1 regulatory pathway to promote flavonoid biosynthesis and salt tolerance in jujube (*Ziziphus jujuba* Mill.)

**DOI:** 10.1186/s12870-026-09254-7

**Published:** 2026-06-12

**Authors:** Cuiping Wen, Zhongtang Wang, Chao Wang, Liguo Qiu, Haiyang Wang, Liying Xiao, Xingang Li

**Affiliations:** 1 College of Life Science, Dezhou University, Dezhou, 253023 China; 2 College of Forestry, Northwest Agriculture and Forestry University, Yangling, Shaanxi 712100 China; 3 Shandong Institute of Pomology, Taian, 271000 China; 4 College of Horticulture and Forestry, Tarim University, Alar, Xinjiang 843300 China

**Keywords:** Jujube (*Ziziphus jujuba* Mill.), Flavonoids, Melatonin, *ZjERF34*, *ZjMYB1*, Salt tolerance

## Abstract

**Background:**

Flavonoids are essential secondary metabolites that contribute to fruit nutritional quality and stress adaptation. Jujube (*Ziziphus jujuba* Mill.) accumulates abundant flavonoids; however, the regulatory mechanisms by which melatonin (MT) modulates flavonoid biosynthesis remain poorly understood.

**Results:**

In this study, exogenous MT treatment markedly promoted flavonoid accumulation in jujube leaves and triggered dynamic changes in endogenous MT levels, implying a regulatory function of MT in this process. Transcriptomic and correlation analyses identified *ZjMYB1* as a key MT-responsive regulator strongly associated with flavonoid accumulation. Functional characterization revealed that silencing *ZjMYB1* significantly decreased flavonoid content and downregulated genes in the phenylpropanoid pathway, whereas its overexpression enhanced flavonoid accumulation in jujube and heterologous systems. *ZjMYB1* directly activated the key structural genes *ZjF3H* and *ZjFLS* by binding to MYB cis-elements in their promoters. Furthermore, the MT-responsive transcription factor *ZjERF34* was identified as an upstream regulator of *ZjMYB1*. *ZjERF34* directly targets the *ZjMYB1* promoter to enhance its transcription, as demonstrated by Y1H, GUS staining, and dual-luciferase assays, establishing a hierarchical ERF–MYB regulatory network. In addition, *ZjMYB1* overexpression significantly enhanced salt tolerance, correlating with increased flavonoid accumulation and upregulation of stress-responsive genes.

**Conclusion:**

Collectively, our findings reveal a MT–ZjERF34–ZjMYB1–ZjF3H/FLS regulatory module that links hormonal signaling with flavonoid biosynthesis and abiotic stress responses, providing new insights and potential molecular targets for improving fruit quality and stress resilience in horticultural crops.

**Supplementary Information:**

The online version contains supplementary material available at 10.1186/s12870-026-09254-7.

## Introduction

As a perennial woody fruit tree in the *Rhamnaceae* family, jujube (*Ziziphus jujuba* Mill.) possesses considerable medicinal and economic importance. Jujube is rich in both primary and secondary metabolites, including polysaccharides, amino acids, flavonoids, triterpenoids, and alkaloids, which makes it a prime example of “medicinal and edible homology” [1, 2]. The leaves, seeds, and fruits of jujube are rich in flavonoid compounds, including catechin, rutin, and quercetin, which contribute to both plant defense and human health. Flavonoids, ubiquitous plant secondary metabolites, are key determinants of nutritional quality and functional properties. In fruits and vegetables, flavonoids not only determine sensory attributes such as color and taste but also play a crucial role in maintaining antioxidant capacity and stress-related metabolic balance [3, 4]. Flavonoid biosynthesis is tightly regulated at the transcriptional level, particularly under environmental stresses. Genes involved in flavonoid biosynthesis are transcriptionally upregulated under various types of environmental stress [5–7], and they play a crucial role in mitigating the accumulation of reactive oxygen species (ROS), thereby enhancing plant stress tolerance through their involvement in environmental responses and biological processes [8]. Several key enzymes involved in flavonoid biosynthesis, including those encoded by *CHS*,* CHI*,* F3’H*, and *DFR*, have been characterized in *Arabidopsis thaliana*, and these data will aid studies of flavonoid biosynthesis in other plants. Additionally, investigations in diverse plant species, including apple, pear, and rice, have revealed key structural genes responsible for flavonoid production, thereby advancing our understanding of this biosynthetic pathway [9, 10]. Nevertheless, flavonoid accumulation in jujube is highly sensitive to environmental conditions, and abiotic stresses such as salinity can disrupt metabolic balance, highlighting the need to understand regulatory mechanisms that coordinate stress tolerance with flavonoid biosynthesis.

Melatonin (N-acetyl-5-methoxytryptamine), a widely distributed indoleamine in plants, animals, and microorganisms, has emerged as a key modulator of secondary metabolism and stress responses [11]. Studies have demonstrated that exogenously applied melatonin can increase flavonoid content while enhancing antioxidant potential in a wide range of plants. For example, in Vitis vinifera, melatonin stimulates the biosynthesis of flavonols and flavanols, particularly catechins [12], while in crabapple leaves, it induces anthocyanin accumulation by upregulating both biosynthetic genes and associated transcription factors [13, 14]. The role of melatonin in plant stress tolerance has been extensively studied in various plant species. Evidence from an increasing number of studies indicates that endogenous melatonin levels rise in plants under abiotic stresses such as cadmium toxicity, high light intensity, low temperatures, and elevated temperatures [15]. Exogenous application of melatonin has been demonstrated to improve plant tolerance to salinity, cadmium, and drought stresses [16, 17]. Melatonin treatment leads to the transcriptional upregulation of stress-responsive genes, including *WRKY1*, *DREB2*, and various MYB transcription factors [18]. Furthermore, melatonin has been shown to have positive effects on the accumulation of soluble solids and lycopene in *Solanum lycopersicum* [19], as well as enhance the synthesis of secondary metabolites in citrus [20]. Recent studies also indicate that melatonin promotes the synthesis of secondary metabolites, especially under stress conditions. For example, exogenous melatonin application regulates the flavonoid content in apples, alleviating the effects of salt stress [21], and foliar application of melatonin increases the total phenolic content in garden thyme leaves under saline conditions [22]. These findings collectively indicate that melatonin plays a crucial role in regulating plant secondary metabolism and stress resistance by orchestrating various physiological and biological processes. Nevertheless, the effect of melatonin on the accumulation of different flavonoid compounds in jujube, as well as the underlying regulatory mechanisms, remains largely unexplored.

Evidence suggests that melatonin-mediated regulation of secondary metabolism may involve crosstalk with ethylene signaling. In tomato and grape, melatonin treatment enhances ethylene biosynthesis by upregulating ACC synthase and ethylene signaling components, thereby modulating fruit ripening and secondary metabolite accumulation [23, 24]. In dragon fruit, ethylene treatment markedly increased the expression of genes involved in the phenylalanine metabolic pathway and enhanced the activities of key enzymes, resulting in elevated accumulation of phenolic compounds [25]. In addition, ethylene-responsive factors (ERFs) are known to integrate hormonal and stress-related signals and participate in the regulation of secondary metabolism [26, 27]. In strawberry, *FaERF9* has been shown to regulate the biosynthesis of 4-hydroxy-2,5-dimethyl-3(2 H)-furanone, a key contributor to strawberry aroma [28]. These observations imply that melatonin-mediated regulation of secondary metabolism may involve ethylene signaling and its downstream transcriptional regulators. However, how melatonin signaling is transduced through transcription factors to modulate flavonoid biosynthesis in jujube has not yet been clearly elucidated.

The biosynthesis of flavonoids is tightly controlled by a complex transcriptional regulatory network, in which MYB transcription factors play a central role. Numerous studies have demonstrated that MYB proteins directly regulate key structural genes in the flavonoid biosynthetic pathway, thereby determining flavonoid composition and content. In *Arabidopsis thaliana*, MYB transcription factors function as key regulators of flavonoid biosynthesis [29]. In tobacco, *NtMYB12a* and *NtMYB12b* both participate in regulating flavonoid biosynthesis, while *NtMYB12a* additionally suppresses fatty acid accumulation in leaves and seeds [30]. *MdMYB1* has also been implicated in regulating anthocyanin accumulation in response to high light exposure [31]. Furthermore, overexpression of MYBs has been shown to enhance the salt stress tolerance of plants. In *Dendrobium catenatum*, overexpression of *DcMYB30* significantly enhances flavonoid accumulation, thereby improving the abiotic stress resilience of plants [32]. In soybeans, *GmMYB12* has been shown to regulate flavonoid accumulation, and its overexpression confers improved salt tolerance to plants [33].

In this study, we investigated the role of melatonin in regulating flavonoid accumulation and salt stress tolerance in jujube. By integrating transcriptomic analysis, molecular assays, and functional validation in both jujube and heterologous systems, we identified a melatonin-responsive ERF-MYB regulatory pathway that links melatonin signaling to flavonoid biosynthesis. Our findings provide new insights into the hormonal regulation of functional compounds in fruit crops and offer valuable genetic targets for improving flavonoid content, stress resilience, and nutritional quality of jujube and other horticultural crops.

## Results

### Exogenous melatonin promotes flavonoid accumulation and modulates endogenous melatonin levels in jujube

**Fig. 1:**
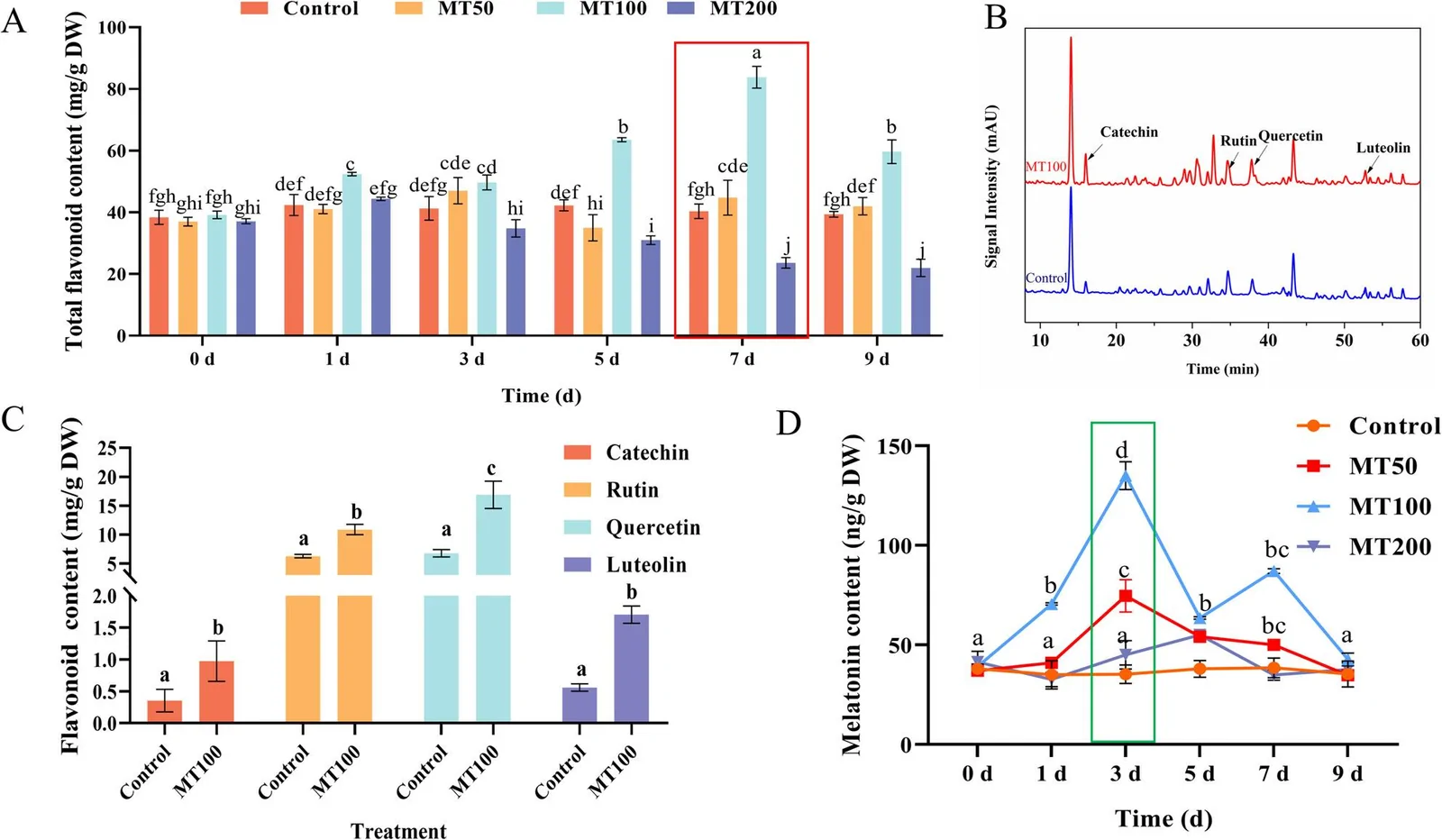
Melatonin induces the accumulation of flavonoids in jujube leaves. **A** The effect of melatonin treatment on the total flavonoid content. **B** Melatonin induces the accumulation of four major flavonoid compounds. **C** Liquid chromatogram of flavonoid compounds. **D** Melatonin content. Different letters (a–i) indicate significant differences according to one-way ANOVA (*p* < 0.05). Error bars indicate mean ± SE

To determine the mechanism by which exogenous melatonin regulates the accumulation of flavonoids, we first conducted a qualitative and quantitative analysis of flavonoid compounds in the leaves of jujube seedlings using HPLC. The seedlings received treatments with varying concentrations of melatonin (50, 100, and 200 µM), and leaf samples were collected at different time points for measurements of the total flavonoid content. The total flavonoid content was two-fold higher in the 100 µM melatonin treatment (83.82 mg/g DW) compared with the control after 7 days of treatment. However, no significant changes in the flavonoid content were observed in the 50 µM melatonin treatment. Treatment with 200 µM melatonin for 3 days induced varying degrees of stress and inhibited flavonoid biosynthesis to some extent. The content of the key flavonoid compounds, catechins, rutin, quercetin, and luteolin, was significantly higher under 100 µM melatonin than in the control after 7 days of treatment (Fig. 1A). Specifically, the content of quercetin and rutin was approximately 173% and 249% higher, respectively, in the melatonin treatment than in the control (Fig. 1B, C). Overall, these results demonstrate that melatonin can induce the accumulation of flavonoid compounds in jujube seedlings.

Exogenous melatonin application also altered endogenous melatonin levels in jujube leaves. Under 100 µM melatonin treatment, endogenous melatonin content rapidly increased and reached a peak at 3 days (135.1 ng/g DW), followed by a gradual decline. A similar trend was observed under 50 µM treatment, where endogenous melatonin levels peaked at 3 days (74.6 ng/g DW) and subsequently decreased (Figure. 1D). Notably, exogenous melatonin treatment promoted the accumulation of both flavonoids and endogenous melatonin in jujube. The early and rapid increase in endogenous melatonin preceded the rise in flavonoid content, suggesting that endogenous melatonin may function as a signaling molecule involved in the induction of flavonoid biosynthesis. 

### Transcriptome profiling reveals differential gene expression and pathway enrichment under melatonin treatment

**Fig. 2:**
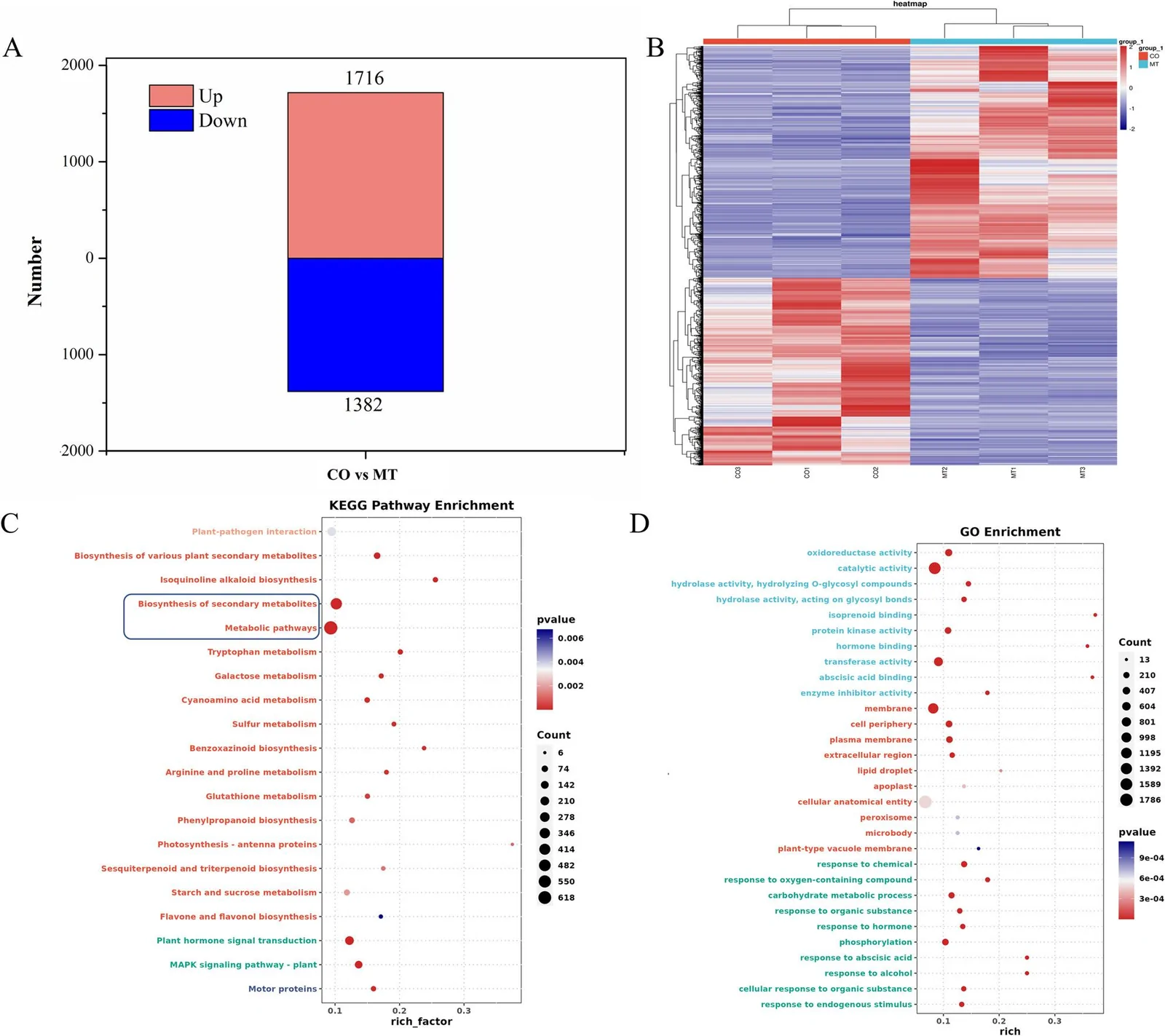
DEGs and KEGG enrichment analyses. **A**, **B** Number of up- and downregulated DEGs. **C** KEGG pathways of the DEGs. **D** GO enrichment of the DEGs

To further explore the molecular mechanism of flavonoid biosynthesis after melatonin treatment, we conducted transcriptome sequencing analysis on the leaves of the jujube plants under the optimal melatonin treatment conditions (100 µM, 7 days). The treatment group (MT1-3) showed a significant separation from the control group (CO1-3) (Fig. 2A). Comparison between CO and MT identified 1,716 upregulated and 1,382 downregulated DEGs (Fig. 2B; Table S7). KEGG pathway enrichment analysis of the DEGs revealed significant enrichment in several metabolic pathways, such as flavonoid biosynthesis, terpenoid biosynthesis, and fatty acid metabolism (Fig. 2C). Concurrently, GO annotation categorized the DEGs into the three standard functional categories: cellular component, biological process, and molecular function (Fig. 2D). 

### Identification of melatonin-responsive transcription factors associated with flavonoid pathway regulation

**Fig. 3:**
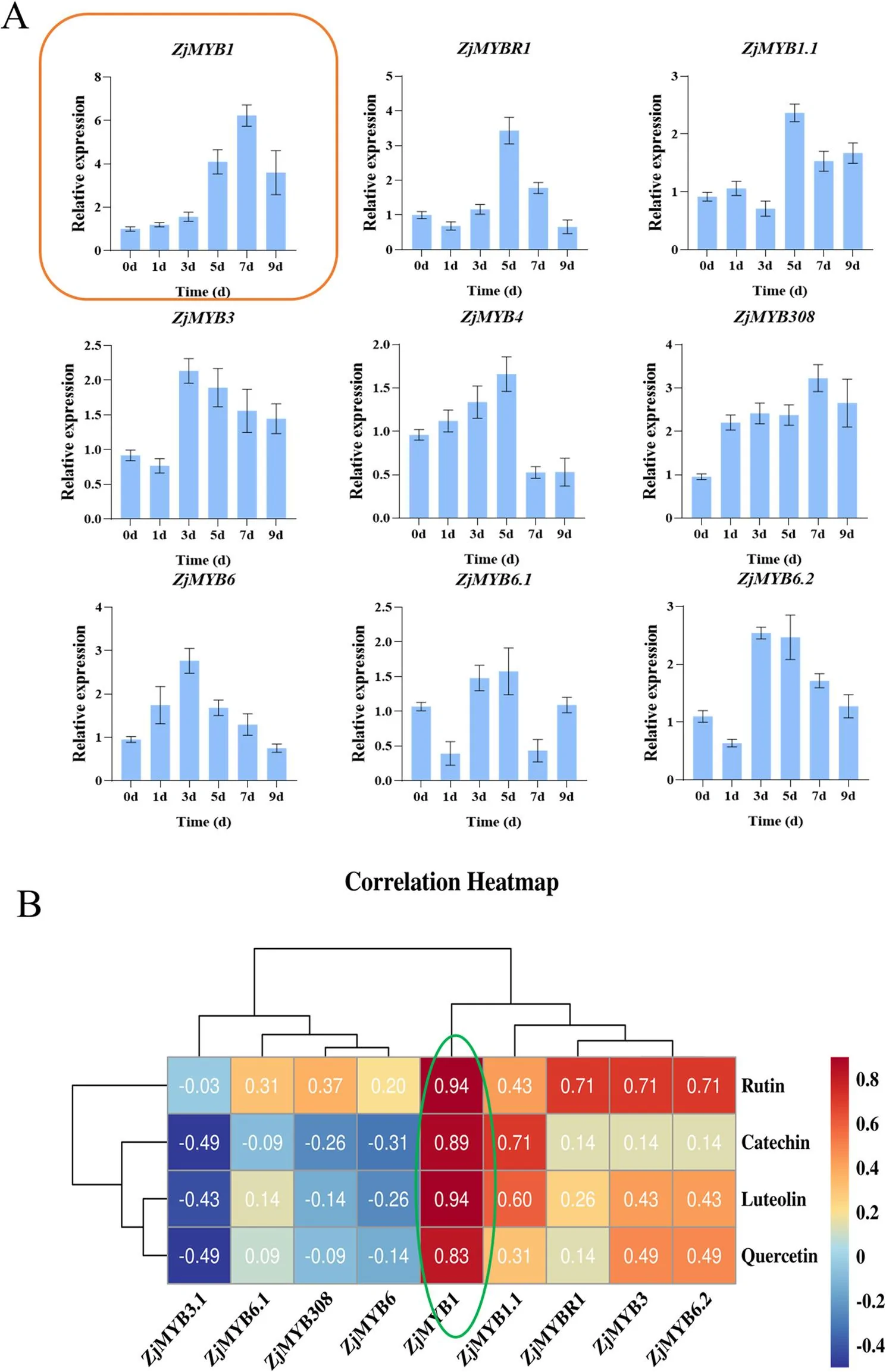
Screening of ZjMYB1 transcription factor **a** The expression of candidate transcription factor MYB induced by melatonin. **B** Correlation analysis of MYB gene expression induced by melatonin and the content of flavonoid compounds. Different letters (a–c) indicate significant differences according to one-way ANOVA (*p* < 0.05)

To further identify the key transcription factors involved in the response to melatonin induction and promote flavonoid accumulation, we analyzed transcriptome data from our previous study and selected nine candidate transcription factors that may be involved in flavonoid biosynthesis (Table S3). To identify putative flavonoid-related MYB transcription factors in jujube, we performed a BLASTP search using known *Glycine max* MYB protein sequences as queries against the annotated jujube proteome (NCBI Accession: GCA_00183579.2). The search applied a stringent e-value cutoff of ≤ 10^− 5^. Candidate TFs were subsequently screened based on their transcript levels and functional annotations, as detailed in Table S3. Subsequently, we extracted total RNA from the leaves of jujube seedlings treated with melatonin for 7 days and measured the expression levels of these transcription factors using qRT-PCR (Fig. 3A, Table S4). We found that the expression of *ZjMYBR1*,* ZjMYB1.1*,* ZjMYB4*, and *ZjMYB6.1* significantly increased after 5 days of induction and decreased thereafter. The expression of *ZjMYB3*,* ZjMYB6.2*, and *ZjMYB6* significantly increased after 3 days of treatment, and that of *ZjMYB1* and *ZjMYB308* significantly increased after 7 days of melatonin treatment; *ZjMYB1* and *ZjMYB308* expression was 6-fold and 3-fold higher, respectively, after 7 days of melatonin treatment compared with the control. To further identify the key transcription factors, we performed a correlation analysis of the expression patterns of these factors and the accumulation of key flavonoid compounds (Fig. 3B). The expression pattern of *ZjMYB1* was most strongly correlated with the flavonoid accumulation pattern. Therefore, we propose that *ZjMYB1* is a key candidate transcription factor involved in flavonoid biosynthesis and highly responsive to melatonin.

### Phylogenetic tree and subcellular localization analysis of *ZjMYB1*

**Fig. 4:**
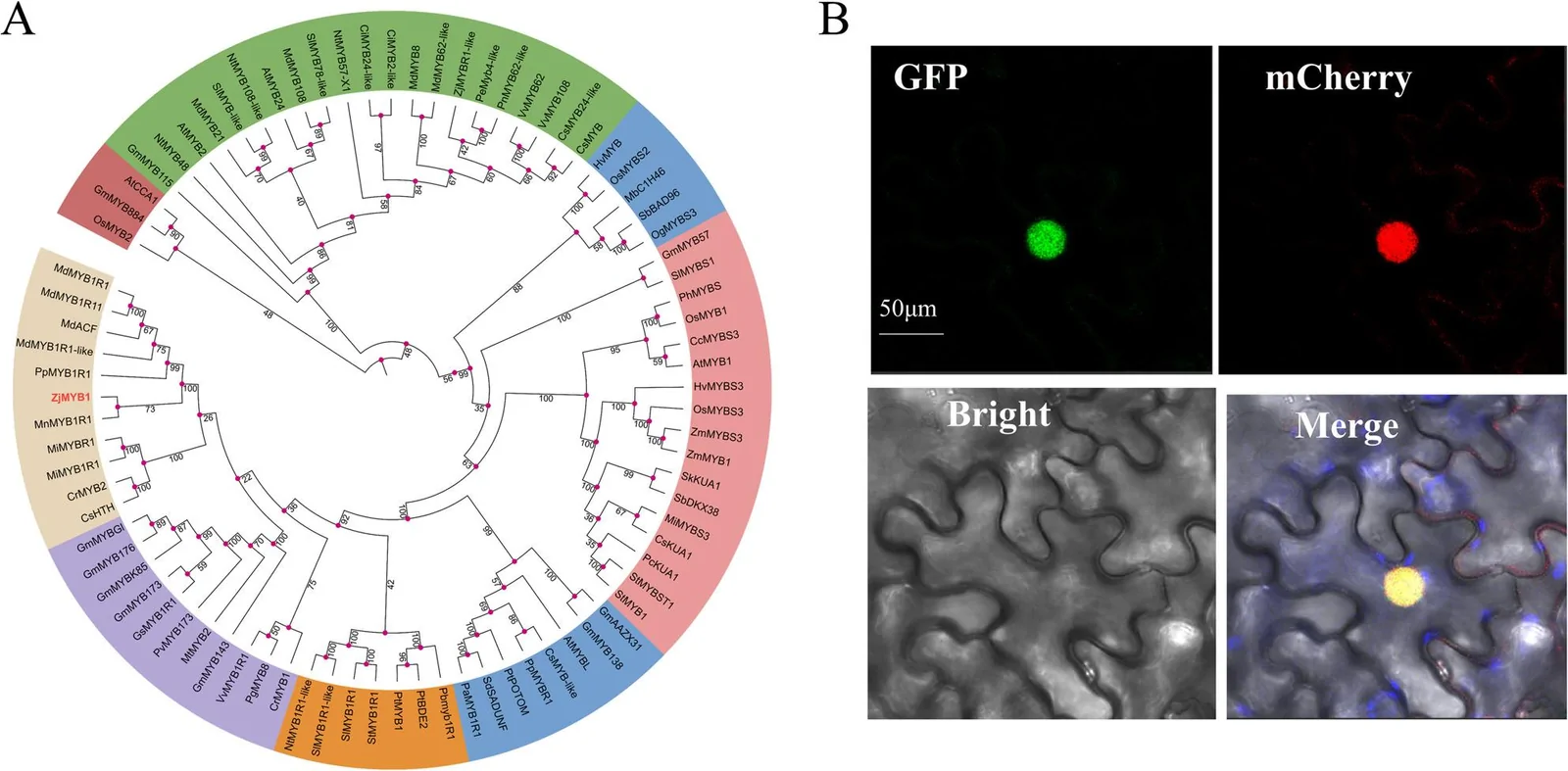
Phylogenetic tree analysis and subcellular localization. **A **Phylogenetic relationship of ZjMYB1 with other plant MYBs. **B** Subcellular localization of ZjMYB1. Scale bar, 50 μm

The *ZjMYB1*gene possesses an ORF spanning 945 bp, which translates into a protein with a deduced length of 315 amino acids. Phylogenetic analysis showed that *ZjMYB1* is highly similar to Group MYBs from *Morus notabilis* (MnMYB1R1), *Malus domestica* (MdMYB1R1), *Prunus persica* (PpMYB1R1), *Mangifera indica* (MiMYB1R1), *Catharanthus roseus* (CrMYB2), and *Citrus sinensis* (CsHTH). Multiple sequence alignments indicated that *ZjMYB1* shares the highest sequence identity with MnMYB1R1, MdMYB1R1, and PpMYB1R1 (73%), followed by MiMYB1R1 and CrMYB2 (26%) (Fig. 4A; Table S3). To investigate its cellular function, we constructed a *ZjMYB1*-GFP fusion expression vector and performed transient transformation of it into tobacco. The GFP-tagged *ZjMYB1* protein was localized to the nucleus after 48 to 60 h (Fig. 4B).

### *ZjMYB1* regulates flavonoid biosynthesis by modulating the expression of structural genes in jujube

**Fig. 5:**
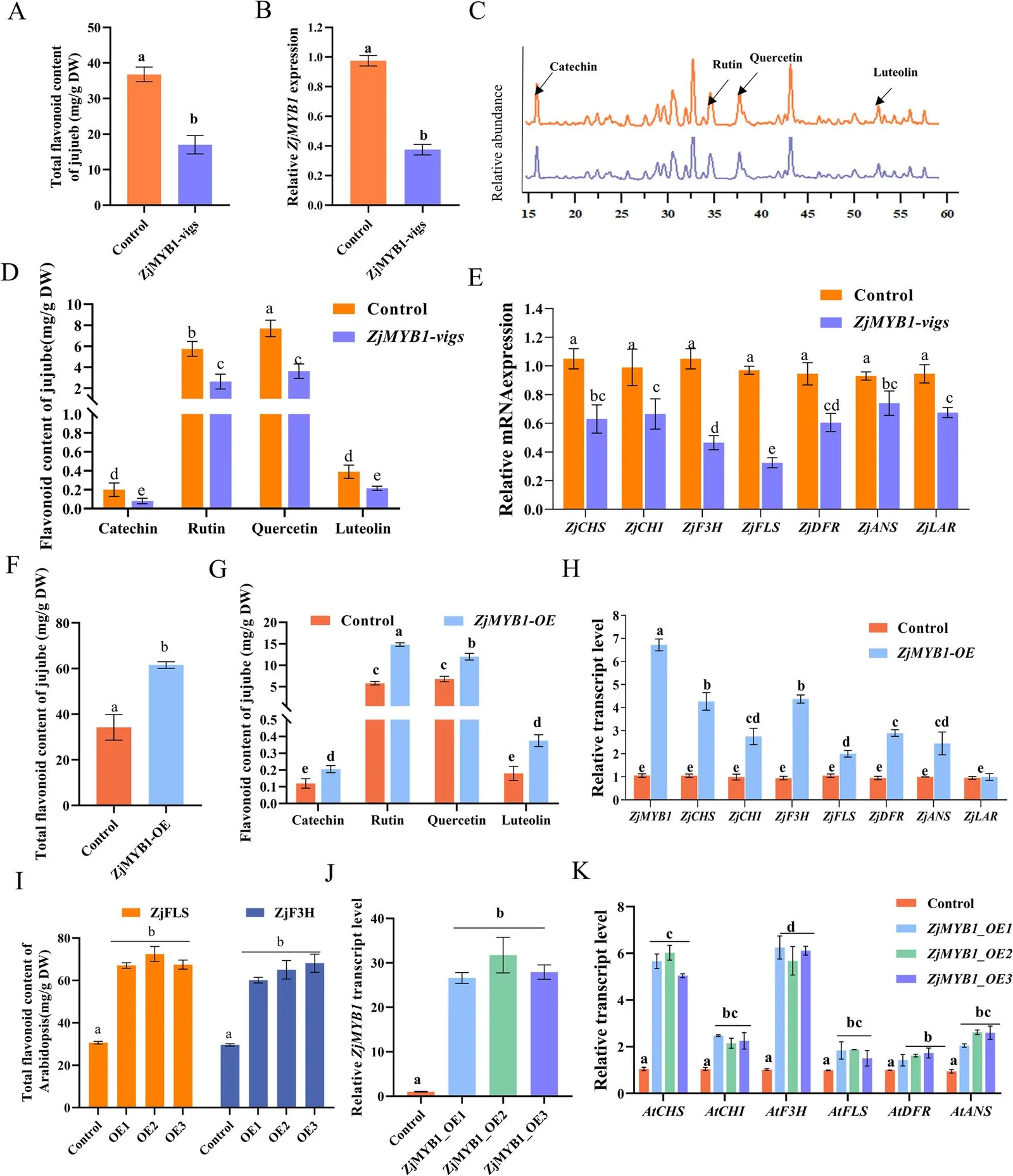
Functional characterization of ZjMYB1 in flavonoid biosynthesis. **A** Total flavonoid content. **B** RT-qPCR analysis of ZjMYB1 expression. **C** Chromatograms showing relative flavonoid content in ZjMYB1-silenced lines compared with controls. **D** The content of four flavonoids. **E** Expression levels of flavonoid pathway genes. **F**, **G** Effect of transient overexpression of ZjMYB1 on flavonoid content. **H** Relative levels of ZjMYB1 and flavonoid pathway genes in control and ZjMYB1-OE overexpression lines. **I** Total flavonoid content changes in OE-ZjMYB1 leaves compared with control. **J**, **K** Expression of ZjMYB1and flavonoid pathway genes in transgenic lines of Arabidopsis thaliana. Different letters (a–d) indicate significant differences according to one-way ANOVA (*p* < 0.05)

To explore the potential role of *ZjMYB1* in flavonoid biosynthesis, we first investigated the effects of *ZjMYB1* silencing on flavonoid accumulation and related gene expression in jujube leaves. Given that *ZjMYB1* can be induced by melatonin, and melatonin enhances the accumulation of flavonoid compounds, we hypothesize that *ZjMYB1* may influence the synthesis of flavonoids, which are important secondary metabolites in jujube plants. To investigate the role of *ZjMYB1* in flavonoid biosynthesis, *ZjMYB1* was silenced in jujube leaves using VIGS. RT-qPCR analysis showed that *ZjMYB1* transcript levels were reduced by approximately 65% compared with the empty vector control (Fig. 5B). HPLC analysis further revealed a significant 56% reduction in total flavonoid content following *ZjMYB1* silencing (Fig. 5A). Moreover, the accumulation of individual flavonoid compounds, including catechin, rutin, quercetin, and luteolin, was significantly lower under melatonin treatment compared with the control. The most substantial reductions were observed for catechin and luteolin (approximately 56% and 58%, respectively), followed by quercetin (approximately 51%) and rutin (approximately 47%) (Fig. 5D). These findings were further confirmed by liquid chromatography (Fig. 5C). Given the reduced flavonoid content due to *ZjMYB1* silencing, we next examined the expression of genes involved in the flavonoid biosynthesis pathway (Table S6). RT-qPCR analysis showed that several key genes in the flavonoid pathway were significantly downregulated in the *ZjMYB1*-VIGS samples compared with the control. Among the core flavonoid biosynthesis genes, the expression of *ZjCHS* decreased by approximately 27%, and the expression of *ZjCHI* decreased by approximately 32%. For genes encoding enzymes involved in flavonoid pathway intermediates, the expression of *ZjDFR* and *ZjANS* decreased by approximately 36% and 20%, respectively. The expression of *ZjF3H* and *ZjFLS* was significantly downregulated by approximately 56% and 70%, respectively. Additionally, the expression of the downstream gene *ZjLAR* was reduced by approximately 28% (Fig. 5E).

To further determine whether *ZjFLS* and *ZjF3H* act as key structural genes mediating flavonoid biosynthesis downstream of *ZjMYB1*, we performed functional characterization analyses. Correlation analysis revealed that the expression levels of *ZjFLS* and *ZjF3H* showed the strongest association with flavonoid accumulation (Figure S2a). Moreover, transient overexpression and silencing experiments in jujube demonstrated that flavonoid content significantly increased and decreased, respectively (Figure S2b, c). Consistently, stable overexpression in *Arabidopsis* also led to a significant increase in total flavonoid content (Figure S2d). Collectively, these results indicate that *ZjFLS* and *ZjF3H* are key structural genes in flavonoid biosynthesis in jujube and likely serve as important downstream targets of *ZjMYB1*.

### Overexpression of *ZjMYB1* enhances flavonoid accumulation and activates biosynthetic pathways

To further validate the regulatory role of *ZjMYB1*, we next investigated whether its overexpression could enhance flavonoid biosynthesis and the expression of related genes by performing a transient overexpression assay in jujube leaves via agroinfiltration. *ZjMYB1* transcript levels driven by the CaMV 35 S promoter were analyzed in leaves 7 days after Agrobacterium tumefaciens infiltration. Gene expression analysis revealed that *ZjMYB1* transcript levels were upregulated by 6.7-fold. The increased transcription levels of key flavonoid biosynthetic genes led to the accumulation of flavonoid compounds in the leaves, and the total flavonoid content increased 1.9-fold to 59.6 mg/g DW (Fig. 5F). Additionally, the accumulation of individual flavonoid compounds showed notable changes, with rutin exhibiting the greatest increase (2.5-fold), followed by luteolin (2.1-fold), quercetin (1.8-fold), and catechin (1.7-fold) (Fig. 5G). Expression analysis showed that most phenylpropanoid pathway genes were upregulated, with *ZjF3H* and *ZjFLS* exhibiting the strongest induction at approximately 4.4- and 4.2-fold, respectively (Fig. 5H), suggesting that these genes may represent key downstream targets of *ZjMYB1*.

To further determine whether this positive regulatory role is conserved across plant species and not limited to jujube, we extended our analysis to heterologous systems. To investigate the positive regulatory role of the *ZjMYB1* transcription factor in the synthesis and accumulation of flavonoids, we overexpressed *ZjMYB1* in *Arabidopsis* and tomato; after RT-PCR and qRT-PCR screening (Figure S3d), three transgenic lines (*ZjMYB1*-OE1, *ZjMYB1*-OE2, *ZjMYB1*-OE3) were obtained. The process for genetically modifying tomato is shown in Figure S2e. In both overexpressing *Arabidopsis* and tomato plants, *ZjMYB1* expression was increased by 29-fold and 35-fold, respectively, compared with the wild-type controls (Fig. 5I, S3a). The total flavonoid content in the transgenic plants was also significantly higher, and it was approximately 2-fold higher in transgenic *Arabidopsis* and tomato leaves than in control plants (Fig. 5G, S3b). To assess whether the expression of flavonoid biosynthetic genes was upregulated in the overexpression plants, we analyzed gene expression levels in overexpression *Arabidopsis*. The transcription levels of the *F3H* and *FLS* genes were approximately 6-fold higher in transgenic plants than in control plants (Fig. 5K). *F3H* and *FLS* expression levels were 4-fold and 5-fold higher in transgenic tomato leaves, respectively, than in the control (Figure S3c). Collectively, these results demonstrate that *ZjMYB1* functions as a positive regulator of flavonoid biosynthesis and strongly suggest that it may promote flavonoid accumulation by directly regulating key structural genes such as *ZjF3H* and *ZjFLS*. 

### *ZjMYB1* directly activates *ZjF3H* and *ZjFLS* by binding to their promoters

**Fig. 6:**
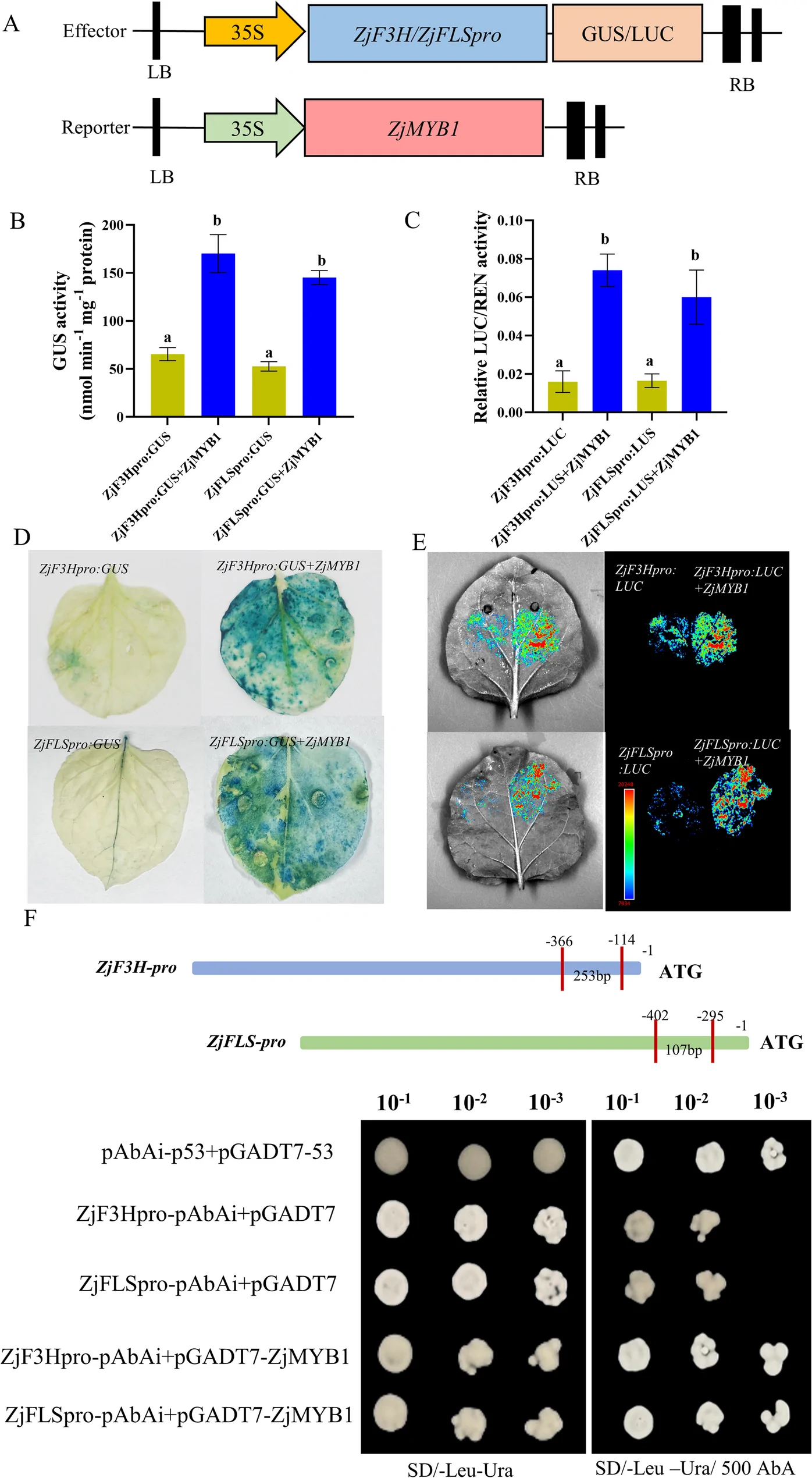
*ZjMYB1* activates the promoters of *ZjF3H* and *ZjFLS*. **A** Schematic of the reporter constructs. **B**, **D** GUS reporter activity detected by histochemical staining and fluorometric assay, respectively. Blue color indicates GUS activity. **C**, **E** Luciferase (LUC) activity detected by imaging and dual-luciferase assays, respectively. The pseudocolor bar indicates luminescence intensity. **F** Yeast one-hybrid assay confirming the direct binding of *ZjMYB1* to the *ZjF3H* and *ZjFLS* promoters. Different letters (a, b) indicate significant differences (*p* < 0.01, one-way ANOVA)

To test the above hypothesis that *ZjMYB1* directly regulates key flavonoid biosynthetic genes, we next investigated whether *ZjMYB1* can activate the promoters of *ZjF3H* and *ZjFLS.* We verified whether *ZjMYB1* can enhance the promoter activity of the key genes *F3H* and *FLS*, thereby increasing flavonoid accumulation (Fig. 6A). We conducted histochemical staining and quantitative protein activity assays. Enhanced promoter activity consequently increased flavonoid accumulation, with *ZjMYB1* elevating *ZjF3H*pro: GUS and *ZjFLS*pro: GUS reporter expression by 2.6- and 2.8-fold, respectively. Consistent with this, GUS staining intensity in *N. benthamiana* leaves reflected the protein activity patterns (Fig. 6B, D). Similarly, in luciferase assays, *ZjMYB1* activated the LUC reporters, with promoter activity quantified via the LUC/REN ratio. Following *ZjMYB1* injection, *ZjF3H*pro: LUC and *ZjFLS*pro: LUC exhibited 4.6-fold and 3.6-fold increases in LUC activity, respectively. The fluorescence intensity in tobacco leaves further confirmed these results (Fig. 6C, E).

Given that overexpression and silencing of *ZjMYB1* respectively upregulated and downregulated the expression of *ZjF3H* and *ZjFLS*, we further examined whether this regulation occurs through direct promoter binding. Promoter sequence analysis revealed the presence of MYB-binding motifs in both *ZjF3H* and *ZjFLS* promoters (Table S5), suggesting that *ZjMYB1* may directly bind to these cis-elements to regulate transcription. To verify this, Y1H assay was performed (Fig. 6F). After transforming the pAbAi-*ZjF3H* and pAbAi-*ZjFLS* constructs into the Y1H system, their activity was inhibited by 500 ng/mL AbA. We expressed *ZjMYB1*, fused with GAL4 AD, in the Y1H system and used the *ZjF3H* and *ZjFLS* promoters fused to the AbAr reporter gene to validate the interaction. The positive control (pAbAi-p53 + pGADT7-53) showed normal growth, while the negative control (empty pGADT7) did not. Importantly, the co-transformation of pGADT7-*ZjMYB1* with *ZjF3H*/*ZjFLS*pro-pAbAi enabled yeast growth under AbA selection, demonstrating that *ZjMYB1* can directly bind to these promoters. These results demonstrate that *ZjMYB1* directly binds to and activates the promoters of *ZjF3H* and *ZjFLS*, thereby promoting flavonoid biosynthesis at the transcriptional level. This finding establishes a direct regulatory link between *ZjMYB1* and key structural genes in the flavonoid pathway, providing a mechanistic basis for its role in flavonoid accumulation.

### *ZjERF34* functions upstream of *ZjMYB1* in the melatonin signaling pathway

**Fig. 7:**
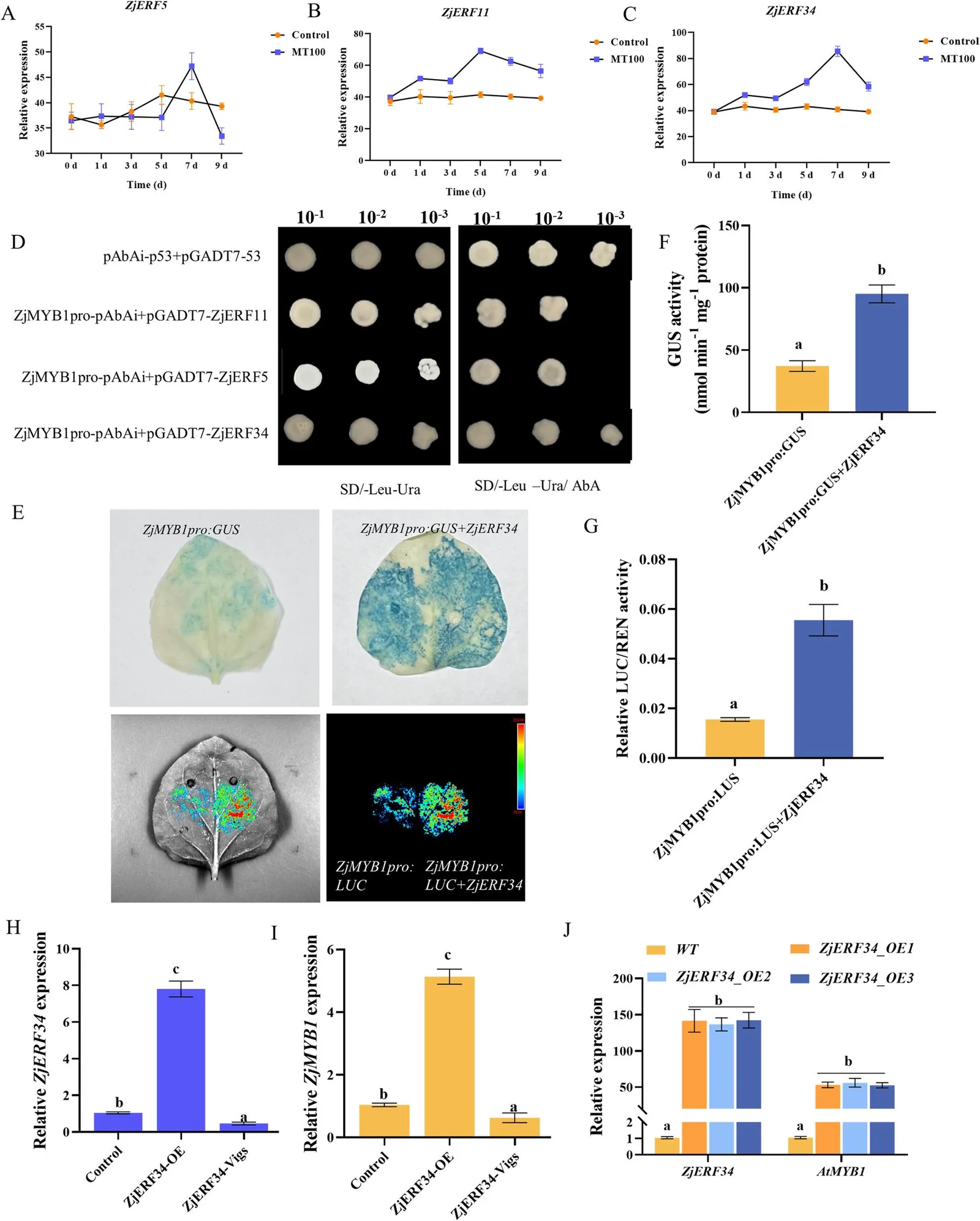
Identification of the ZjERF34 transcription factor and its regulatory effect on ZjMYB1. **A**-**C** Melatonin-induced expression of ERF transcription factors; **D** Yeast one-hybrid screening of ERF transcription factors binding to ZjMYB1; **E**-**G** GUS staining and LUC activity detection. Blue color indicates GUS activity. The pseudocolor bar indicates luminescence intensity. **H**-**I** Effects of ERF34 overexpression and silencing on gene expression; **J** Gene expression levels in overexpressing Arabidopsis plants. Different letters (a-c) indicate significant differences (*p* < 0.05, one-way ANOVA)

Having established that *ZjMYB1* directly activates key flavonoid biosynthetic genes such as *ZjF3H* and *ZjFLS*, we next sought to identify the upstream regulators that control *ZjMYB1* expression, particularly under MT signaling conditions. Given previous evidence that ethylene-responsive factors (ERFs) can be induced by MT and are involved in the regulation of secondary metabolism, we hypothesized that MT-responsive ERFs may function upstream of *ZjMYB1* to modulate its transcription and thereby regulate flavonoid biosynthesis. Using local BLAST analysis, three putative MT-induced ERF transcription factors were identified from the jujube genome: *ZjERF5*, *ZjERF11*, and *ZjERF34*. Following MT treatment, expression analysis revealed that *ZjERF34* was significantly upregulated after 7 days, *ZjERF11* exhibited a marked increase in transcript abundance after 5 days, whereas *ZjERF5* showed only a relatively modest induction (Fig. 7A-C). These results suggest that *ZjERF34* may play a dominant role in MT-mediated transcriptional regulation.

To determine whether these ERFs could regulate *ZjMYB1* expression, yeast one-hybrid assays were performed, which demonstrated that among the three candidates, only *ZjERF34* was able to bind to the GCC-box cis-element within the *ZjMYB1* promoter (Fig. 7D, S4). To validate whether ERF specifically binds to the GCCGCC cis-element, we performed yeast one-hybrid assays with wild-type and mutated baits. The wild-type bait contained three tandem copies of GCCGCC, while the mutant bait contained three tandem copies of GCCAAA where the core motif was disrupted. Yeast cells co-transformed with AD-ERF34 and the wild-type bait grew robustly on selective medium, whereas those co-transformed with AD-ERF34 and the mutant bait failed to grow under the same conditions. These results demonstrate that ZjERF34 specifically binds to the GCCGCC cis-element (Figure S7). This interaction was further validated by GUS staining and dual-luciferase reporter assays, which confirmed that *ZjERF34* binds to the *ZjMYB1* promoter and significantly activates its transcriptional activity (Fig. 7E-G)).

To verify the regulatory role of *ZjERF34* in controlling *ZjMYB1* transcription, *ZjERF34* was transiently overexpressed or silenced in jujube leaves. RT-qPCR analysis showed that transient overexpression of *ZjERF34* significantly increased *ZjMYB1* transcript levels, whereas *ZjERF34* silencing markedly reduced *ZjMYB1* expression (Fig. 7H, I). To assess whether this regulatory relationship is conserved, *ZjERF34* was stably overexpressed in *Arabidopsis* thaliana. Consistently, the expression of *AtMYB1* was significantly upregulated in three independent transgenic lines compared with wild-type plants (Fig. 7J), suggesting that this regulatory module may be evolutionarily conserved. Collectively, these findings indicate that *ZjERF34* directly activates *ZjMYB1* by binding to its promoter. Importantly, when integrated with our previous findings, this study establishes a hierarchical regulatory cascade linking melatonin signaling to flavonoid biosynthesis via the *ZjERF34*–*ZjMYB1*–*ZjF3H*/*FLS* module, providing new insights into the transcriptional network underlying flavonoid accumulation in jujube. 

### *ZjMYB1* enhances salt tolerance through coordinated activation of flavonoid biosynthesis and stress-responsive genes

**Fig. 8:**
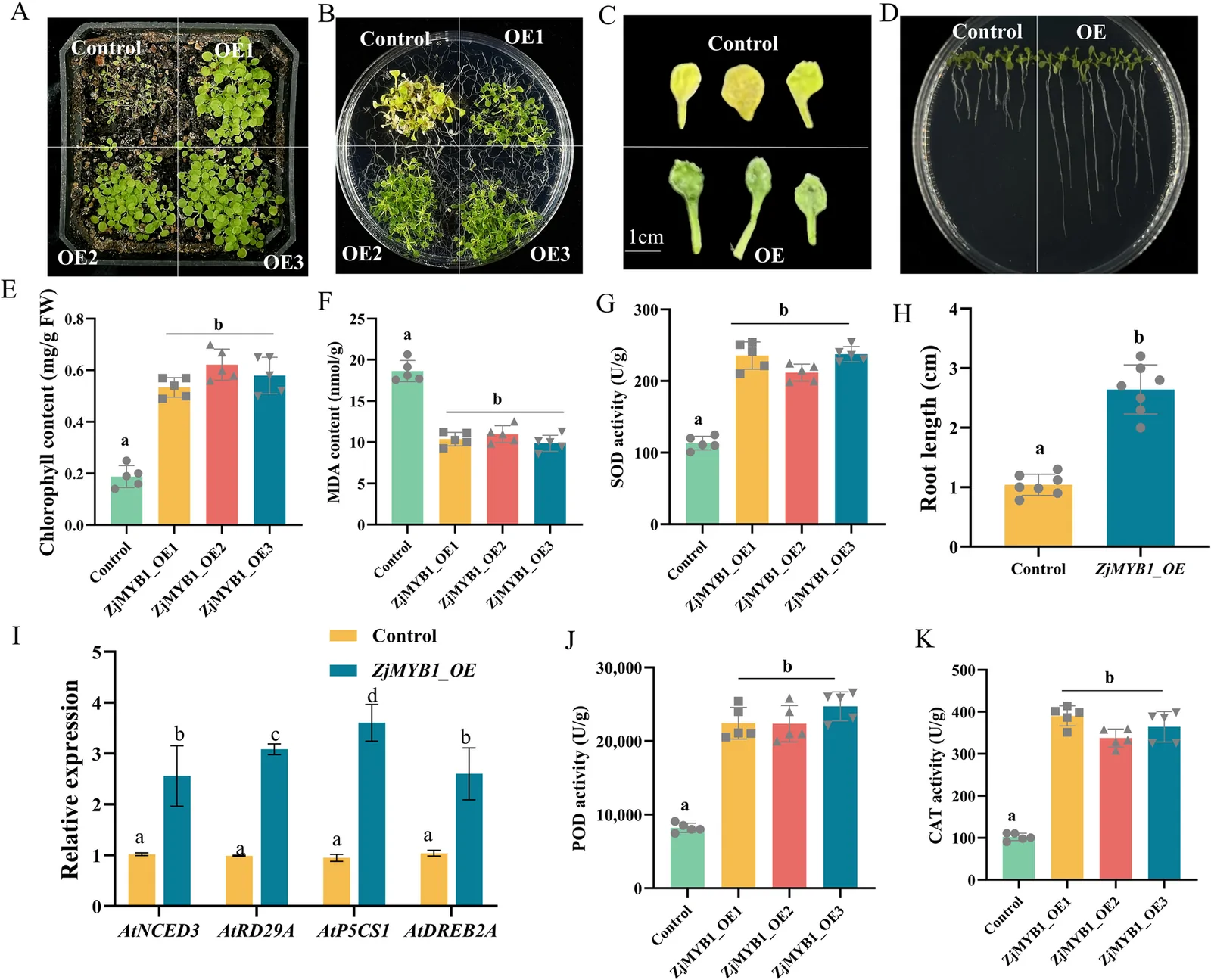
Overexpression of *ZjMYB1* enhances salt tolerance in *Arabidopsis* thaliana. **A**-**C** Phenotypic comparison of *ZjMYB1*-overexpressing (OE) and wild-type (WT) plants under salt stress. **D**, **H** Root length. **E** Chlorophyll content. **F**, **G**, **J**, **K** Levels of MDA and SOD, POD, and CAT. **I** Expression levels of defense-related genes. Data represent mean ± SD (*n* = 5). Different letters indicate significant differences (*p* < 0.05, one-way ANOVA)

Building on the above findings that *ZjMYB1* functions downstream of melatonin signaling to regulate flavonoid biosynthesis, we next investigated whether this regulatory pathway contributes to salt stress tolerance. MT treatment alleviated salt-induced damage in jujube plants, accompanied by increased flavonoid accumulation and significant upregulation of *ZjMYB1* expression (Figure S5), suggesting a role for *ZjMYB1* in stress adaptation. To further evaluate its function, salt stress assays were conducted using *ZjMYB1*-overexpressing *Arabidopsis* and tomato plants. Under 200 mM NaCl treatment, transgenic *Arabidopsis* exhibited higher germination rates, improved growth, and significantly longer roots (approximately 2.5-fold) compared with wild-type plants (Fig. 8A–D, H). Similarly, transgenic tomato plants displayed enhanced tolerance, maintaining greener leaves with reduced wilting symptoms under salt stress (Figure S6a). Physiological analyses revealed that transgenic plants had higher chlorophyll content and lower malondialdehyde (MDA) levels, along with significantly increased activities of antioxidant enzymes, including SOD, POD, and CAT (Fig. 8E–G, J, K; Figure S6). In addition, the expression levels of stress-responsive genes such as *P5CS*,* RD29A*,* DREB2A*, and *NCED3* were markedly upregulated in transgenic plants (Fig. 8I; Figure S6c). Taken together, these results indicate that *ZjMYB1* enhances salt tolerance through coordinated regulation of multiple downstream targets, including flavonoid biosynthetic genes and stress-responsive genes. The precise contribution of flavonoid accumulation to this phenotype requires further investigation. Combined with our previous findings, this study supports a regulatory model in which the MT–*ZjERF34*–*ZjMYB1* module integrates flavonoid biosynthesis with abiotic stress responses.

## Discussion

Flavonoids are key bioactive components contributing to the nutritional quality and functional value of jujube, and their accumulation is highly sensitive to environmental conditions [34]. Salt stress not only restricts plant growth but also disrupts secondary metabolite biosynthesis, thereby affecting the stability of quality-related compounds in horticultural crops. In recent years, melatonin has emerged as an important regulator of plant stress responses and metabolic homeostasis [35], yet its role in coordinating flavonoid accumulation and stress tolerance in fruit crops remains insufficiently understood. In this study, we demonstrate that exogenous melatonin significantly promotes the accumulation of multiple flavonoid compounds in jujube leaves while simultaneously enhancing salt stress tolerance, highlighting its dual role in improving both functional composition and stress resilience.

Previous studies have shown that melatonin can modulate flavonoid biosynthesis in several plant species by upregulating phenylpropanoid pathway genes, thereby enhancing the accumulation of flavonols, anthocyanins, and related metabolite [36]. In *Rosa rugosa*, exogenous melatonin has been shown to upregulate key structural genes involved in flavonoid biosynthesis, including *Rr4CL*, *RrF3H*, and *RrANS*, thereby promoting the accumulation of flavonols (e.g., quercetin) and anthocyanins (e.g., peonidin-3-O-glucoside) [37]. Similarly, in *Cajanus cajan*, melatonin treatment induces the accumulation of various metabolites, primarily flavonoids (e.g., luteolin), which is directly correlated with the upregulation of phenylpropanoid pathway enzyme genes, including *C4H*, *F5H*, and *CHI* [11]. Consistent with these observations, our results indicate that melatonin treatment markedly increases the contents of key flavonoids, including catechin, rutin, quercetin, and luteolin, in jujube. Transcriptomic and correlation analyses further revealed that this melatonin-induced flavonoid accumulation is closely associated with the upregulation of flavonoid biosynthetic genes, particularly *F3H* and *FLS*. These findings suggest that melatonin contributes to the stabilization and enhancement of flavonoid profiles in jujube, which is of direct relevance to maintaining nutritional quality under adverse environmental conditions.

Transcription factors play a central role in integrating hormonal signals with secondary metabolism. MYB transcription factors, in particular, are widely recognized as key regulators of flavonoid biosynthesis across plant species [38]. In this study, *ZjMYB1* was identified as a central regulatory node linking melatonin signaling with flavonoid biosynthesis in jujube. RNAi-mediated silencing of *CsPP2-A1* in cucumber resulted in a significant downregulation of key phenylpropanoid pathway genes (*CsPAL*, *CsCAD*) and flavonoid biosynthetic genes (*CsF3H*, *CsCHS*, *CsFLS*). This led to a decrease in the levels of metabolites such as luteolin and naringenin chalcone, thereby reducing overall flavonoid accumulation [39]. Functional analyses demonstrated that silencing *ZjMYB1* significantly reduced flavonoid accumulation and suppressed the expression of phenylpropanoid pathway genes, whereas overexpression of *ZjMYB1* led to enhanced flavonoid production in jujube as well as in heterologous systems, including *Arabidopsis* thaliana and tomato. These results indicate that *ZjMYB1* functions as a conserved positive regulator of flavonoid biosynthesis and represents a key determinant of flavonoid content stability in plants.

Further mechanistic analyses revealed that *ZjMYB1* directly activates the transcription of *F3H* and *FLS* by binding to MYB cis-elements in their promoters, thereby promoting the metabolic flux toward flavonoid biosynthesis in jujube. In *Bletilla striata*, overexpression of *BsMYB51* directly activates key rate-limiting enzyme genes (*PAL*,* CHS*,* F3H*,* DFR*) in the phenylpropanoid pathway, resulting in the accumulation of flavonoids, anthocyanins, and proanthocyanidins [40]. Likewise, *SbMYB3* from *Astragalus membranaceus* binds to the promoter of *SbFNSII*, a root-specific rate-limiting enzyme, driving the efficient accumulation of medicinal flavonoids such as baicalein and baicalin in roots [41]. These findings establish a direct regulatory link between *ZjMYB1* and flavonoid biosynthesis, providing mechanistic insight into how flavonoid accumulation is transcriptionally controlled in jujube.

Beyond this direct regulation, our study further uncovers an upstream regulatory layer in which *ZjMYB1* is activated by the MT-responsive transcription factor *ZjERF34*. *ZjERF34* directly binds to the promoter of *ZjMYB1* and enhances its transcription, thereby forming an ERF–MYB regulatory cascade. This finding provides a molecular explanation for how MT signaling is transduced into transcriptional activation of flavonoid biosynthesis. Similar interactions between ERF and MYB transcription factors have been reported in *Arabidopsis* thaliana, where ERFs integrate hormonal signals to regulate stress responses [42, 43]. Taken together, our results establish a hierarchical regulatory module, MT–*ZjERF34*–*ZjMYB1*–*ZjF3H*/FLS, which links hormonal signaling to secondary metabolism. From a food science perspective, this module provides a mechanistic framework explaining how environmental signals regulate the accumulation of nutritionally important metabolites in fruit crops.

In addition to its role in flavonoid biosynthesis, *ZjMYB1* overexpression significantly improved salt stress tolerance, as evidenced by enhanced growth performance, higher chlorophyll content, reduced lipid peroxidation, and increased antioxidant enzyme activities under saline conditions, an observation consistent with findings in other plant species [44]. Moreover, *ZjMYB1* overexpression led to the upregulation of stress-responsive genes such as *NCED*,* DREB*,* RD29*, and *P5CS*, indicating that *ZjMYB1* integrates metabolic and stress signaling pathways. These results are in agreement with previous studies indicating that MYB transcription factors enhance abiotic stress tolerance through the regulation of both secondary metabolism and stress-responsive genes [45, 46]. Notably, similar coordination between flavonoid biosynthesis and stress tolerance has been reported for *GbMYB11* in *Ginkgo biloba*, where enhanced flavonol accumulation is associated with improved salt tolerance [47]. Despite these consistent correlations, the causal relationship between flavonoid accumulation per se and the observed stress tolerance requires cautious interpretation. It should be noted that while *ZjMYB1*-mediated flavonoid accumulation consistently correlates with enhanced salt tolerance across multiple transgenic lines and species, the present study does not unequivocally establish flavonoid accumulation as the direct and sole cause of stress tolerance. *ZjMYB1* also activates a suite of classical stress-responsive genes, which are known to contribute independently to osmotic adjustment and stress signaling. Therefore, the salt-tolerant phenotype of *ZjMYB1*-overexpressing plants likely results from a coordinated transcriptional program rather than from flavonoid accumulation alone. Pharmacological inhibition of flavonoid biosynthesis (e.g., using specific inhibitors of chalcone synthase or phenylalanine ammonia-lyase) or genetic disruption of flavonoid pathway genes in *ZjMYB1*-overexpressing backgrounds would be valuable approaches to quantitatively dissect the causal contribution of flavonoids in future studies.

## Conclusion

**Fig. 9:**
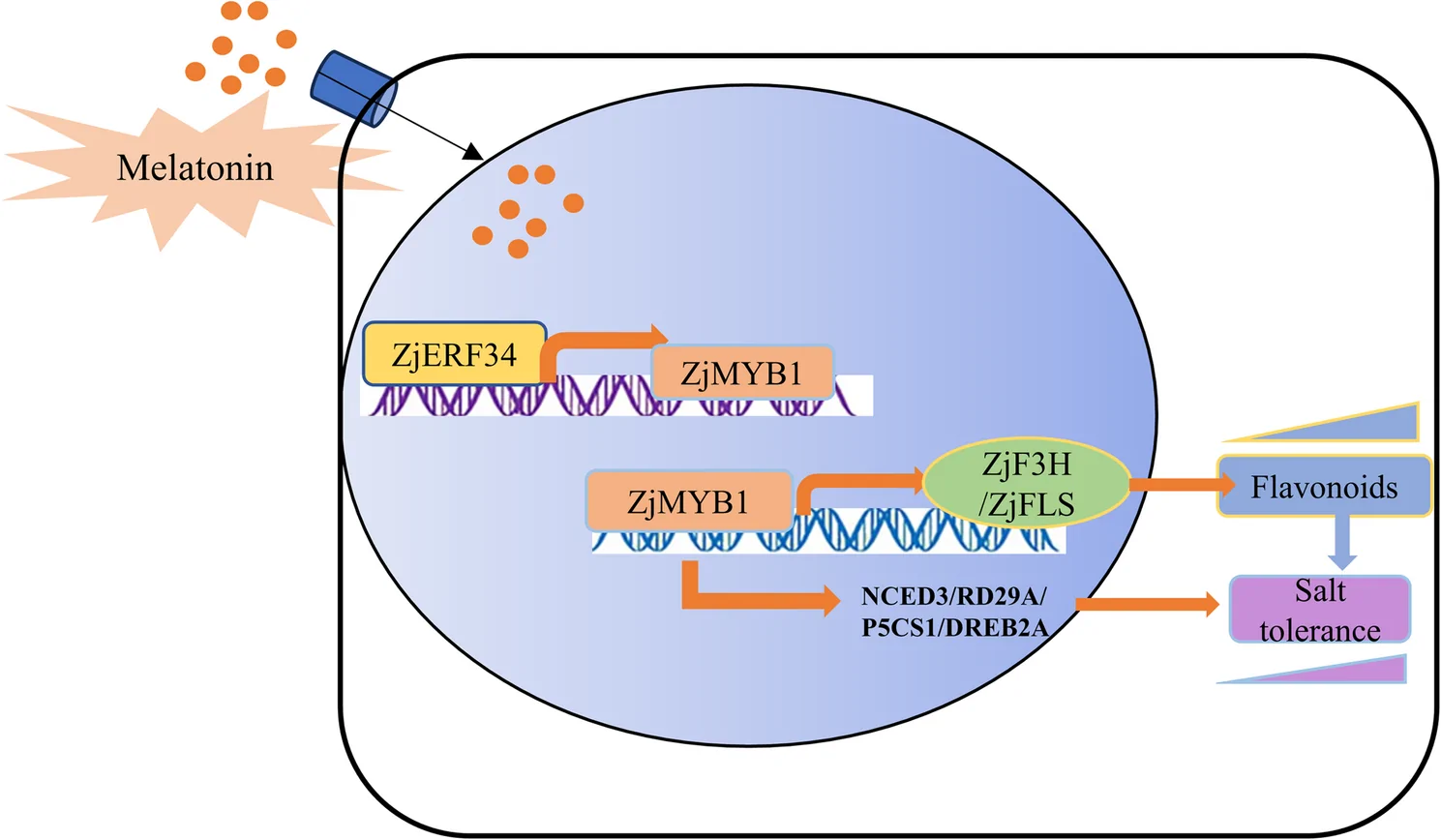
The mechanism model of melatonin-induced flavonoid biosynthesis and improvement of salt tolerance

In this study, we show that melatonin promotes flavonoid accumulation and enhances salt tolerance in jujube (Fig. 9). Mechanistically, *ZjMYB1* functions as a key positive regulator by directly activating *ZjF3H* and *ZjFLS*, while being transcriptionally induced by the melatonin-responsive factor *ZjERF34*, forming a hierarchical regulatory cascade. This MT–*ZjERF34*–*ZjMYB1*–*ZjF3H*/*FLS* module links hormonal signaling with flavonoid biosynthesis and stress responses, with *ZjMYB1* coordinates the expression of multiple downstream targets, including flavonoid biosynthetic genes and stress-responsive genes, which collectively contribute to improved stress adaptation. This work not only advances our understanding of flavonoid regulatory networks in fruit crops but also provides a promising molecular target for improving crop quality and stress tolerance.

## Materials and methods

### Plant materials

Wild jujube seeds were sourced from the experimental station in Qingjian, Yulin, China. All seedlings were maintained in climate-controlled greenhouse at 24℃ with 16/8 h (light/dark) photoperiod. Three-week-old seedlings demonstrating homogeneous growth were selected for experimental use. For melatonin treatment, plants were sprayed three times daily with a 100 µM melatonin solution until runoff was observed; control plants were sprayed with water via the same procedure. Leaves were harvested on days 0, 1, 3, 5, 7, and 9 after treatment, immediately frozen using liquid nitrogen and then placed in storage.

For salt stress treatment, jujube seedlings were foliar-sprayed with 200 mM NaCl, with or without 100 µM melatonin, to assess its potential alleviating effect. For transient assays, *N. benthamiana* was grown in a growth chamber (28 °C, 16/8 h light/dark). Stable transgenic *Arabidopsis* thaliana and Solanum lycopersicum plants were maintained under similar photoperiods at 22–24 °C. All systems were subsequently subjected to salt stress for functional analysis.

### Material extraction and qualitative and quantitative analysis of flavonoids

Flavonoids were extracted and determined using a combination of ultrasonic-assisted extraction and spectrophotometric methods. Briefly, plant samples were powdered and extracted with 70% ethanol under ultrasonic conditions (40 kHz, 60 °C, 30 min). The extracts were filtered and concentrated under reduced pressure, and total flavonoid content was determined using the aluminum chloride colorimetric method with rutin as the standard. The quantification was based on absorbance readings at 510 nm, and the data are presented as milligrams of rutin equivalents per gram of dry weight (mg RE/g DW).

The total flavonoid content (TFC) was determined following the method of Li et al. with minor modifications [48]. For high-performance liquid chromatography (HPLC) analysis of individual flavonoid compounds, a reversed-phase C18 column (4.6 × 250 mm, 5 μm) was used with gradient elution. The mobile phase comprised 0.1% formic acid in water (A) and 0.1% formic acid in acetonitrile (B), with a flow rate of 1.0 mL min⁻¹, column temperature of 30 °C, and detection at 280 nm for flavones/flavonols. The identification of individual flavonoids was achieved by aligning their retention times and UV spectra with those of authentic standards. For quantification, an external calibration method exhibiting excellent linearity (R² > 0.999) was employed.

### Melatonin extraction and determination

The leaf sample (0.2 g) was extracted by ultrasonication for 30 min in the dark using pre-chilled methanol containing 0.1% BHT. The mixture was centrifuged at 4 °C (12,000 rpm for 10 min), and the supernatant was collected and passed through a 0.22 μm organic membrane filter prior to HPLC. Chromatographic separation was carried out using a methanol–water mixture (6:4, v/v) as the mobile phase in isocratic mode, at a flow rate of 1.0 mL min⁻¹. The column temperature was maintained at 30 °C, with detection performed at 278 nm and an injection volume of 10 µL. Quantification was based on an external standard calibration curve generated from a series of melatonin standard solutions. Melatonin was identified by its retention time and quantified according to peak area.

### Gene expression and transcriptome analysis

After screening out the most suitable melatonin treatment concentration (100 µM) and time (7 days), the samples under specific treatment conditions were subjected to transcriptome sequencing. Total RNA was isolated with a commercial kit, and its quality was verified by spectrophotometry (NanoDrop) and agarose gel electrophoresis. High-quality RNA served as the template for first-strand cDNA synthesis using a reverse transcriptase kit with oligo(dT) and random primers. Subsequent qPCR analyses, performed in triplicate with SYBR Green Master Mix, normalized gene expression to *ZjUBQ1*and *ZjUBQ2* and calculated levels via the 2^−ΔΔCT^ method (Table S1). For transcriptome sequencing, qualified RNA libraries were sequenced on an Illumina HiSeq 4000 platform. The resulting clean reads were aligned to the jujube reference genome using HISAT2 for gene identification, with expression quantified by FPKM. Screening for DEGs was performed with DESeq2 by applying a cutoff of |log2(Fold Change)| ≥ 1 and an adjusted p-value < 0.01. Finally, DEGs were subjected to functional enrichment analysis of GO terms and KEGG pathways.

### Subcellular localization analysis

The CDs of gene was cloned into a pCambia2300-GFP vector using specific primers with restriction sites (BamHI and SalI). The primers are shown in Table S2. The fusion construct and the empty vector were transformed into GV3101 and transiently expressed in *N. benthamiana* leaves. Fluorescence signals were captured using a confocal laser-scanning microscope (TCS SP8 SR; Leica Zeiss, Germany).

### Phylogenetic tree analysis

Phylogenetic analysis of the MYB transcription factors involved in regulating flavonoid biosynthesis and responses to phytohormones such as melatonin was performed using MEGA v.6 with default settings and the neighbor-joining method (Table S3). Branch support was evaluated using 1,000 bootstrap replicates (accessed on 8 July 2025).

### Vector construction and plant transformation

The pTRV2-key gene construct was generated by cloning a gene-specific fragment, amplified from jujube leaf cDNA, into the pTRV2 vector. For overexpression, the open reading frame (ORF) of the target gene was inserted into the pC2300 vector under the control of the CaMV 35 S promoter (Table S2; Figure S1). Agrobacterium tumefaciens GV3101 was transformed with both constructs. The flavonoid content in jujube leaves was quantified 10 days following transient transformation. For stable transformation, the plasmid was introduced into *Arabidopsis* and tomato via the floral-dip and leaf disc methods, respectively [49, 50]. Transgenic lines were confirmed by PCR and qRT-PCR, with *ZjUBQ1* and *ZjUBQ2* (jujube), *AtActin* (*Arabidopsis*), and *SlActin* (tomato) serving as reference genes.

### Analysis of physiological indicators under salt stress treatment

One-week-old transgenic and wild-type plants were subjected to salt stress treatment with 200 mM NaCl, using water as a control. Following phenotypic observation, all plant samples were snap-frozen in liquid nitrogen and stored at -80 °C. The activities of key antioxidant enzymes, including superoxide dismutase (SOD), peroxidase (POD), and catalase (CAT), along with the concentration of malondialdehyde (MDA), were quantified using commercial assay kits. Total chlorophyll was quantified from NaCl-treated and control plants of tomato and *Arabidopsis thaliana* using the method of Wang et al. [51].

### Glucuronidase and luciferase activity assays

The ~ 2.0 kb promoter sequences of genes were cloned into the pC0390GUS and pGreenII 0800-LUC vectors, respectively, while the CDs of target gene was inserted into the pGreen62-SK vector (Table S2). All constructs were co-transformed into Agrobacterium tumefaciens GV3101 (Weidi, Shanghai) harboring the pSoup helper plasmid. The bacterial suspensions containing the promoter-reporter and effector constructs were mixed and infiltrated into the abaxial side of tobacco leaves. After 48–60 h, the leaves were harvested for glucuronidase (GUS) staining and luciferase (LUC) activity assays using a commercial kit (Beyotime, Nanjing).

### Yeast one-hybrid (Y1H) assay

The promoter fragments of the target genes containing the core cis-acting elements were cloned into the pAbAi plasmid (Table S2). These constructs were linearized and integrated into the Y1HGold yeast genome. The successfully transformed strains were screened on SD/-Ura medium supplemented with AbA. The coding sequences of the TFs were cloned into the pGADT7 vector and transformed into Y1HGold cells harboring pAbAi-genes. The empty pGADT7-P53 and pGADT7 vectors were used as positive and negative controls, respectively.

### Yeast one-hybrid assay with site-directed mutagenesis

Bait constructs were generated by inserting three tandem copies of wild-type (GCCGCC) or mutated (GCCAAA) sequences into the pAbAi vector. Linearized bait constructs were integrated into the Y1HGold yeast. pGADT7-*ZjERF34* or empty pGADT7 (negative control) was transformed into yeast strains containing wild-type or mutated baits. The successfully transformed strains were screened on SD/-Ura medium supplemented with AbA.

### Statistical analyses

Differences in flavonoid content were assessed by one-way analysis of variance (ANOVA) with Duncan’s multiple comparison test. These statistical analyses were conducted using SPSS software. Data visualization and statistical charting were conducted using GraphPad Prism (version 8.0.2), Tbtools, and the OmicShare Tools online platform (https://www.omicshare.com/tools/; accessed on 10 July 2025), with a significance threshold defined as *p* < 0.05 for all statistical comparisons.

## Supplementary Information

Supplementary Material 1: Figure S1. pCambia2300-GFP and pTRV2 derived vector maps used for subcellular localization, overexpression (a) and VIGS (b) studies, respectively. Figure S2. Screening and identification of key synthetic genes for flavonoids in jujube. (a) Correlation analysis; (b, c) Instantaneous silencing and overexpression of ZjF3H and ZjFLS on the nutrition of monomeric flavonoid compounds; (d) The effect of stable overexpressed Arabidopsis on total flavonoids. Figure S3. Effects of overexpression of ZjMYB1 on the expression level of flavonoid synthesis pathway and total flavonoids in tomato. (a) Relative levels of ZjMYB1 and flavonoid pathway genes in control and ZjMYB1-OE overexpression lines. (b) Total flavonoid content changes in OE-ZjMYB1 leaves compared with control. (c) Expression of ZjMYB1 and flavonoid pathway genes in transgenic lines of tomato. (d) RT-PCR verification of positive Arabidopsis and tomato plants. (e) The process of converting tomatoes using the leaf disc method. Figure S4.The specific sequence of the ZjMYB1 promoter, GUS/LUC reporter and effector systems. Figure S5. MT alleviates salt stress in jujube plants. (a) Both NaCl and NaCl + MT increase the expression of ZjMYB1; (b) NaCl and NaCl + MT promote the accumulation of total flavonoids; (c) The state of jujube plants under NaCl and NaCl + MT. Figure S6. Effect of ZjMYB1 overexpression on defense-related genes and salt stress in tomato.(a) Growth phenotypes of overexpressed ZjMYB1 tomato and control under salt stress; (b) The difference of chlorophyll content between overexpressed and control lines; (c) Expression levels of defense related-genes; (d-g) The analysis of MDA content and SOD, POD, and CAT activities in overexpressed and control lines. Figure S7. ZjERF34 specifically binds to the GCCGCC sequence of the ZjMYB1 promoter.

Supplementary Material 2: Table S1. The primers of real-time qPCR analysis. Table S2. Primers for expression vector construction. Table S3 Referenced MYBs used to construct phylogenetic tree. Table S4. Annotated information about the TFs in jujube. Table S5. The promoter sequences. Table S6. Annotated information about the flavonoid synthesis genes. Table S7. The List of differentially expressed genes after melatonin treatment.

## Data Availability

The jujube reference genome analyzed during the current study is available in the NCBI Genome database under accession number GCA_00183579.2. The raw transcriptome data supporting the conclusions of this article are available in the NCBI SRA repository under accession number PRJNA1466623. All other data generated during this study are included in this published article and its supplementary information files. No new materials were generated during this study.
