# Supplementary material for: Melatonin activates the ZjERF34-ZjMYB1 regulatory pathway to promote flavonoid biosynthesis and salt tolerance in jujube (Ziziphus jujuba Mill.)
Source: BMC Plant Biol. 2026 Jun 12;26:1414. doi: 10.1186/s12870-026-09254-7 (PMC13488009; doi:10.1186/s12870-026-09254-7)
Supplement: Supplementary file 1 [file 12870_2026_9254_MOESM1_ESM.pdf]

Supplementary Information

Manuscript Title: Melatonin activates the ZjERF34-ZjMYB1 regulatory pathway to promote flavonoid biosynthesis and salt tolerance in jujube (*Ziziphus jujuba* Mill.)

Cuiping Wen[1,2*], Zhongtang Wang[3], Chao Wang[2], Liguo Qiu[1], Haiyang Wang[1], Liying Xiao[1], Xingang Li[,2,4 *]

1 College of Life Science, Dezhou University, Dezhou 253023, China

2 College of Forestry, Northwest Agriculture and Forestry University, Yangling 712100, Shaanxi, China

3 Shandong Institute of Pomology, Taian 271000, China

4 College of Horticulture and Forestry, Tarim University, Alar 843300, Xinjiang, China

* Corresponding author

E-mail address: xingangle@nwsuaf.edu.cn; wencuiping913@163.com

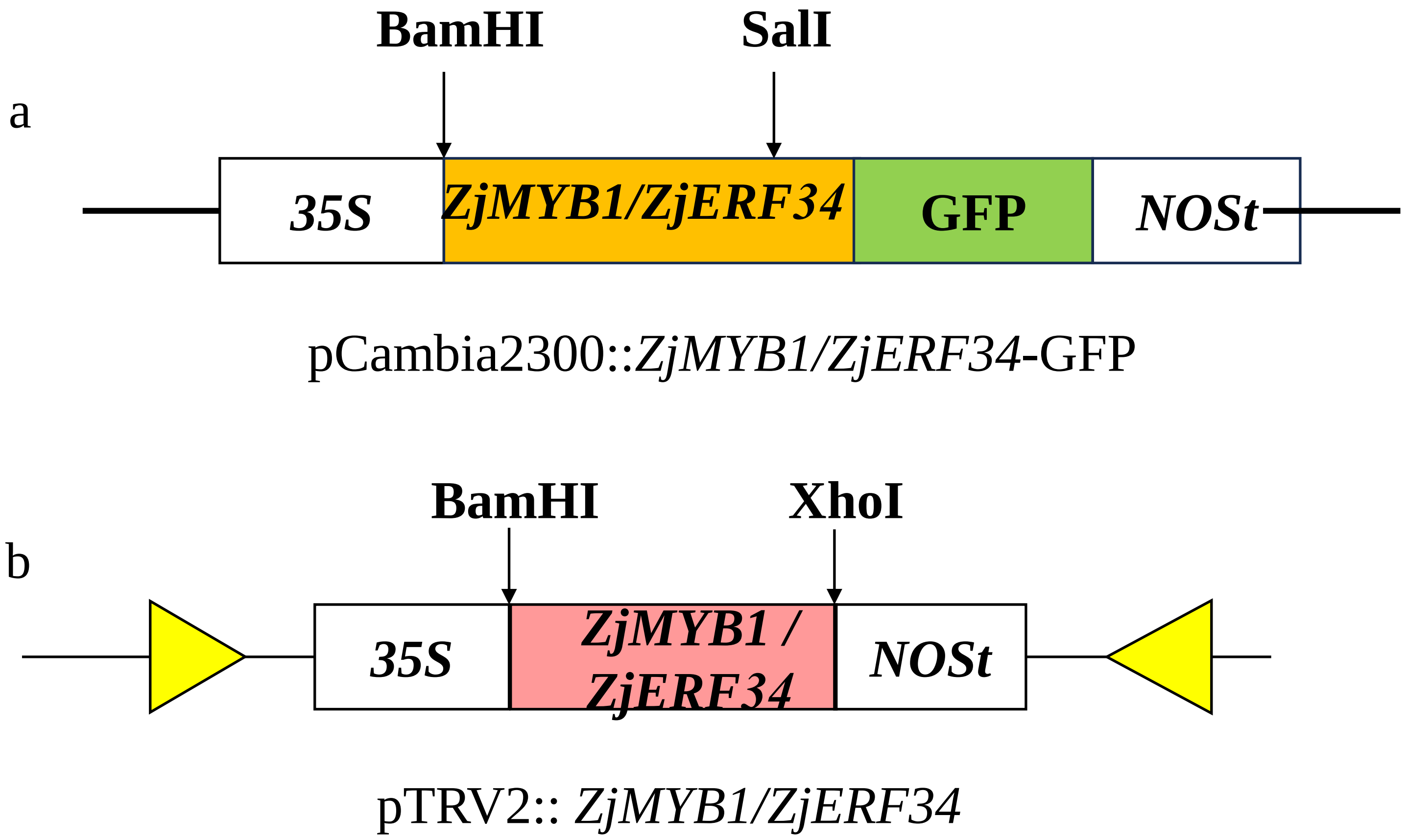

Figure S1 pCambia2300-GFP and pTRV2 derived vector maps used for subcellular localization, overexpression (a) and VIGS (b) studies, respectively.

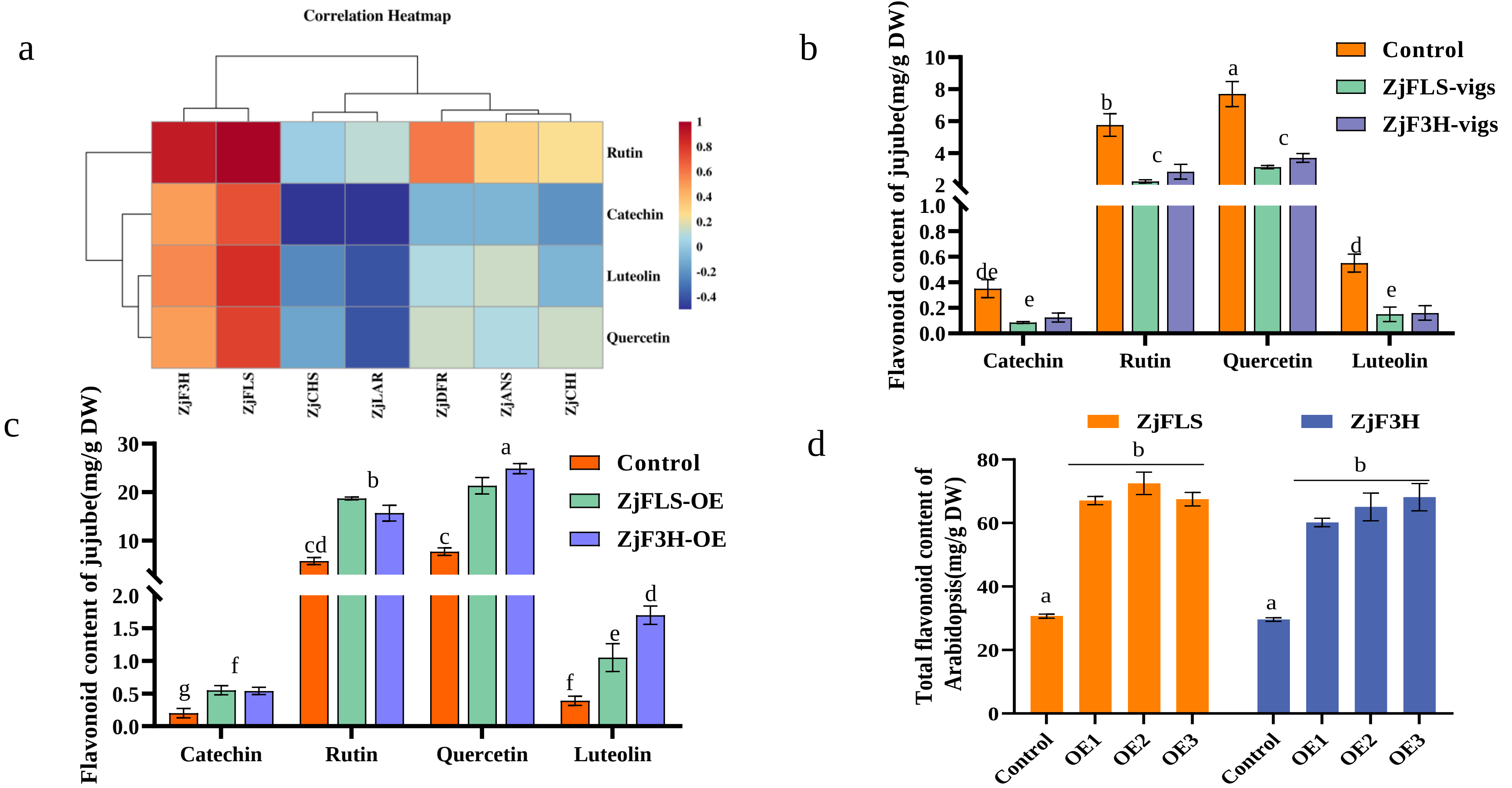

Figure S2. Screening and identification of key synthetic genes for flavonoids in jujube. (a) Correlation analysis; (b, c) Instantaneous silencing and overexpression of *ZjF3H* and *ZjFLS* on the nutrition of monomeric flavonoid compounds; (d) The effect of stable overexpressed *Arabidopsis* on total flavonoids. Different letters (a–g) indicate significant differences according to one-way ANOVA ($p<0.05$). Error bars indicate mean ± SE.

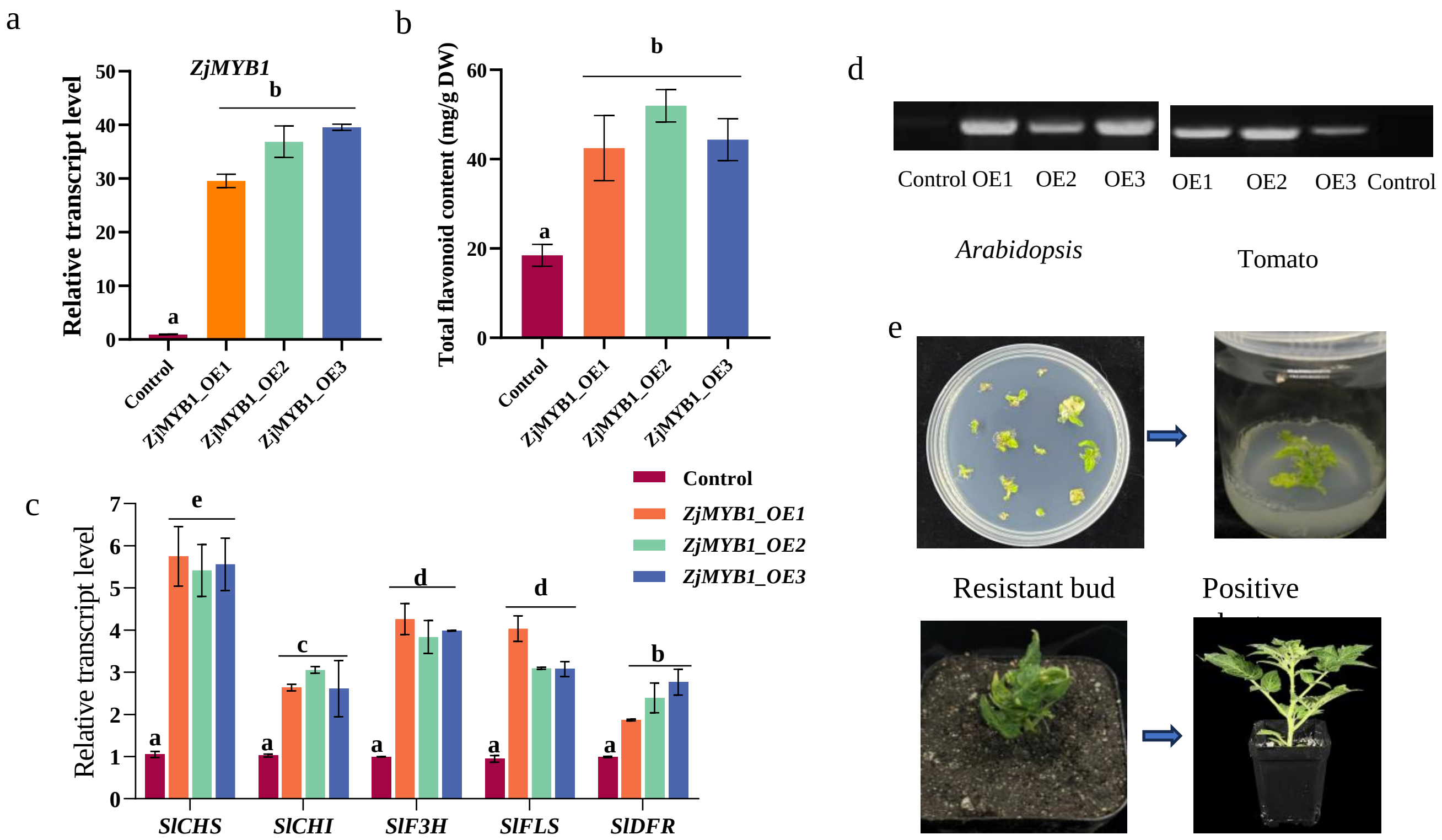

Figure S3. Effects of overexpression of ZjMYB1 on the expression level of flavonoid synthesis pathway and total flavonoids in tomato. (a) Relative levels of ZjMYB1 and flavonoid pathway genes in control and ZjMYB1-OE overexpression lines. (b) Total flavonoid content changes in OE-ZjMYB1 leaves compared with control. (c) Expression of ZjMYB1 and flavonoid pathway genes in transgenic lines of tomato. (d) RT-PCR verification of positive Arabidopsis and tomato plants. (e) The process of converting tomatoes using the leaf disc method. Different letters (a–d) indicate significant differences according to one-way ANOVA ($p<0.05$). Error bars indicate mean ± SE.

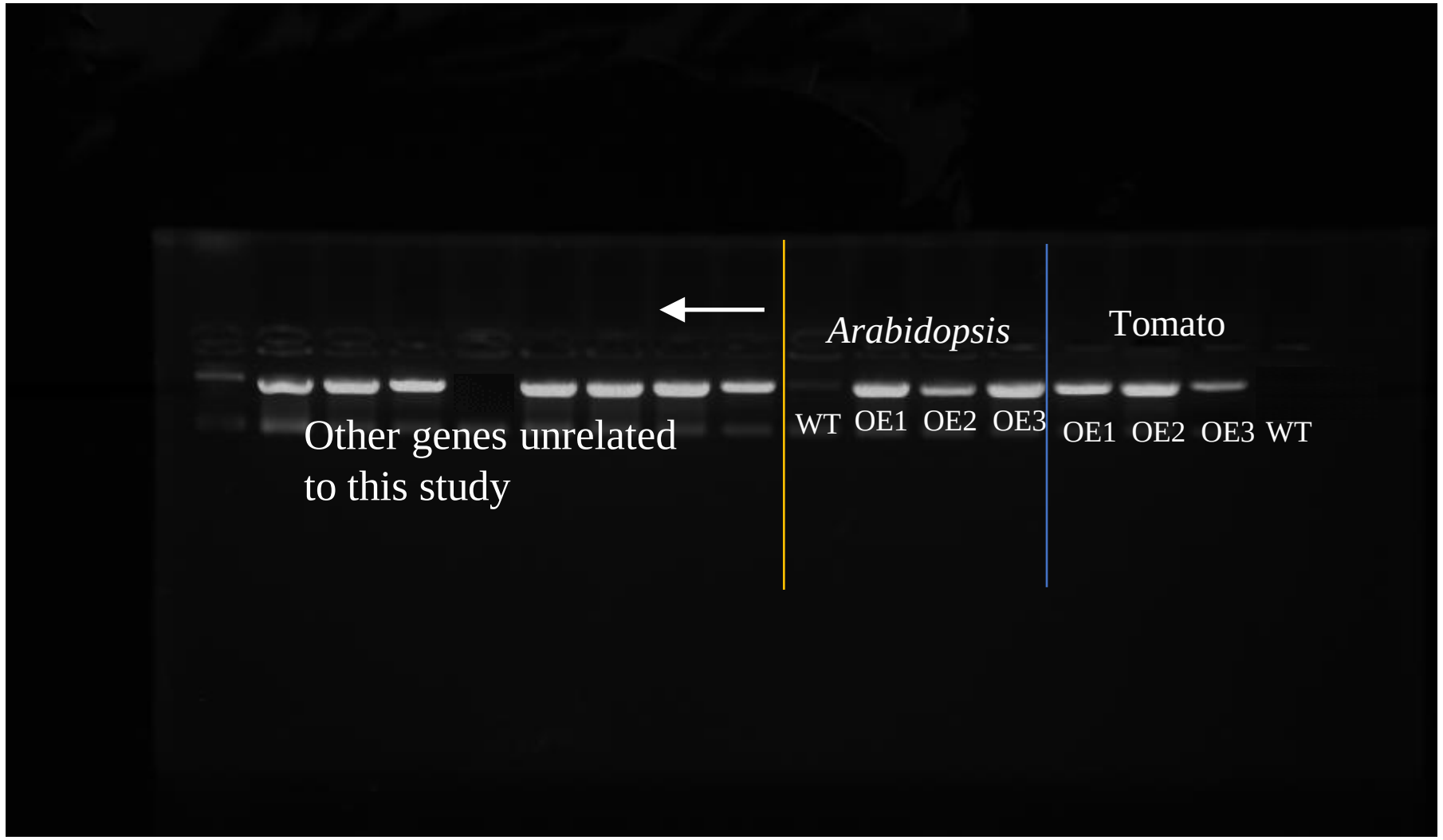

Original electrophoresis pattern

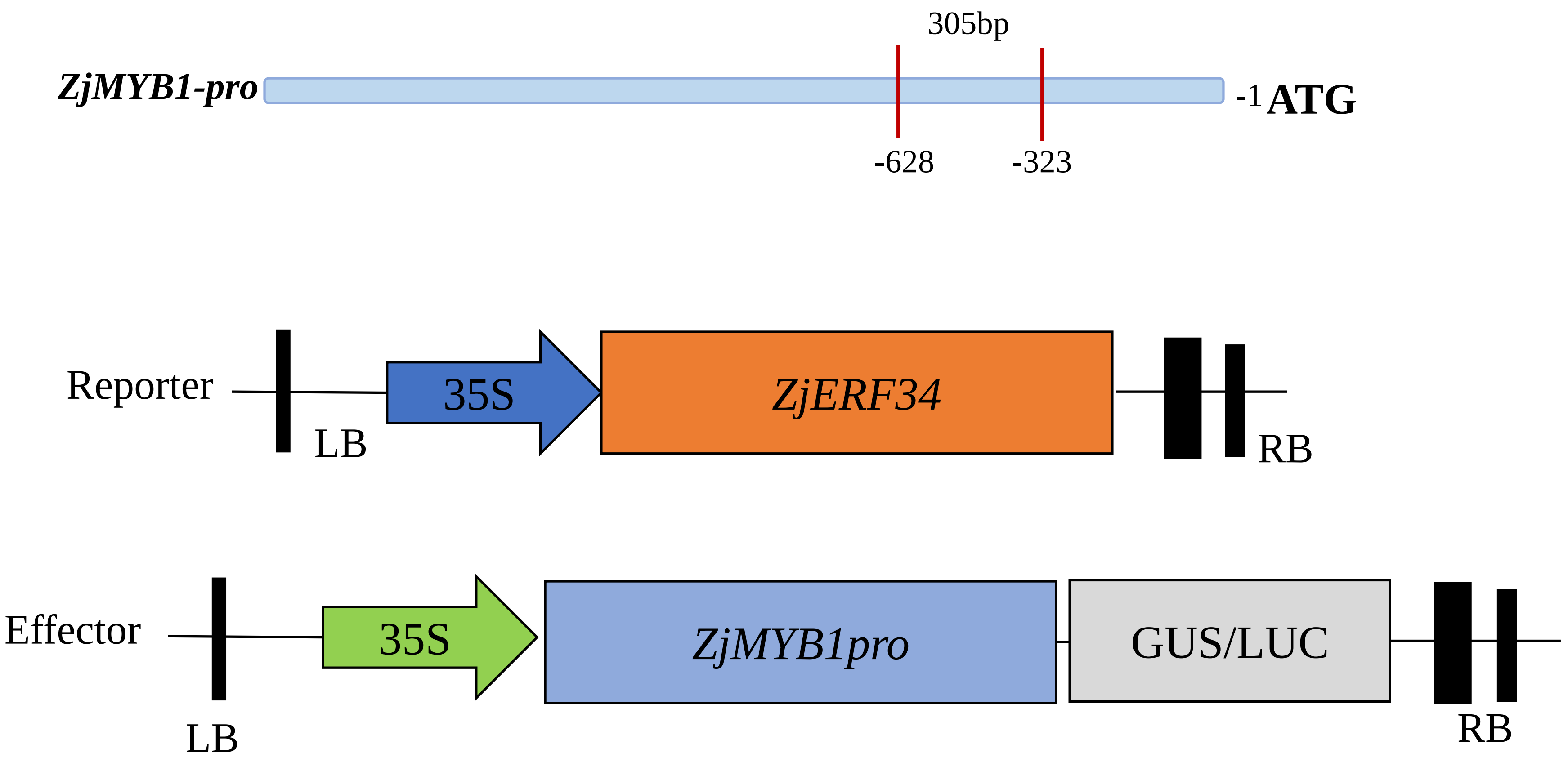

Figure S4.The specific sequence of the ZjMYB1 promoter, GUS/LUC reporter and effector systems

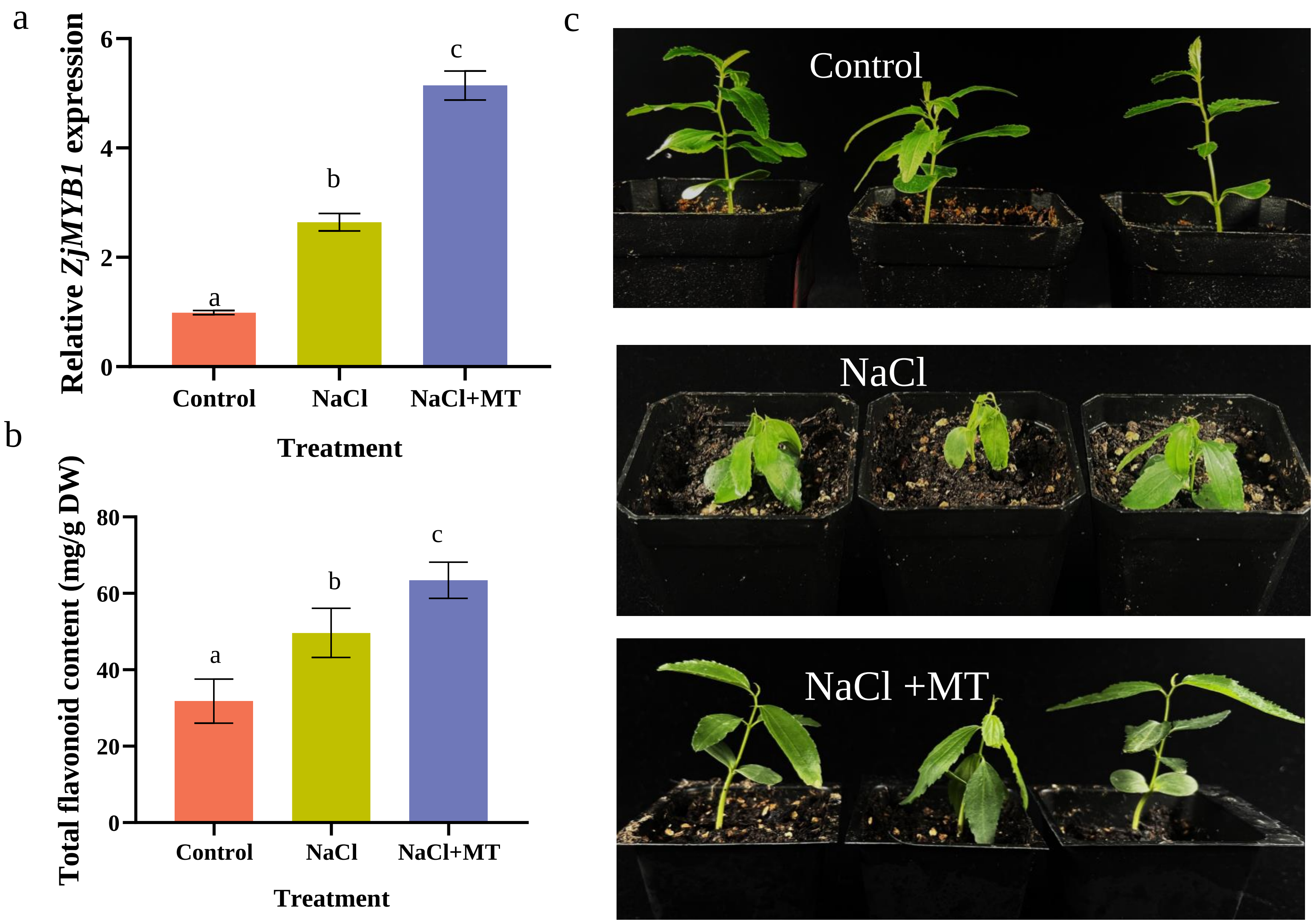

Figure S5. MT alleviates salt stress in jujube plants. (a) Both NaCl and NaCl + MT increase the expression of ZjMYB1; (b) NaCl and NaCl + MT promote the accumulation of total flavonoids; (c) The state of jujube plants under NaCl and NaCl + MT.

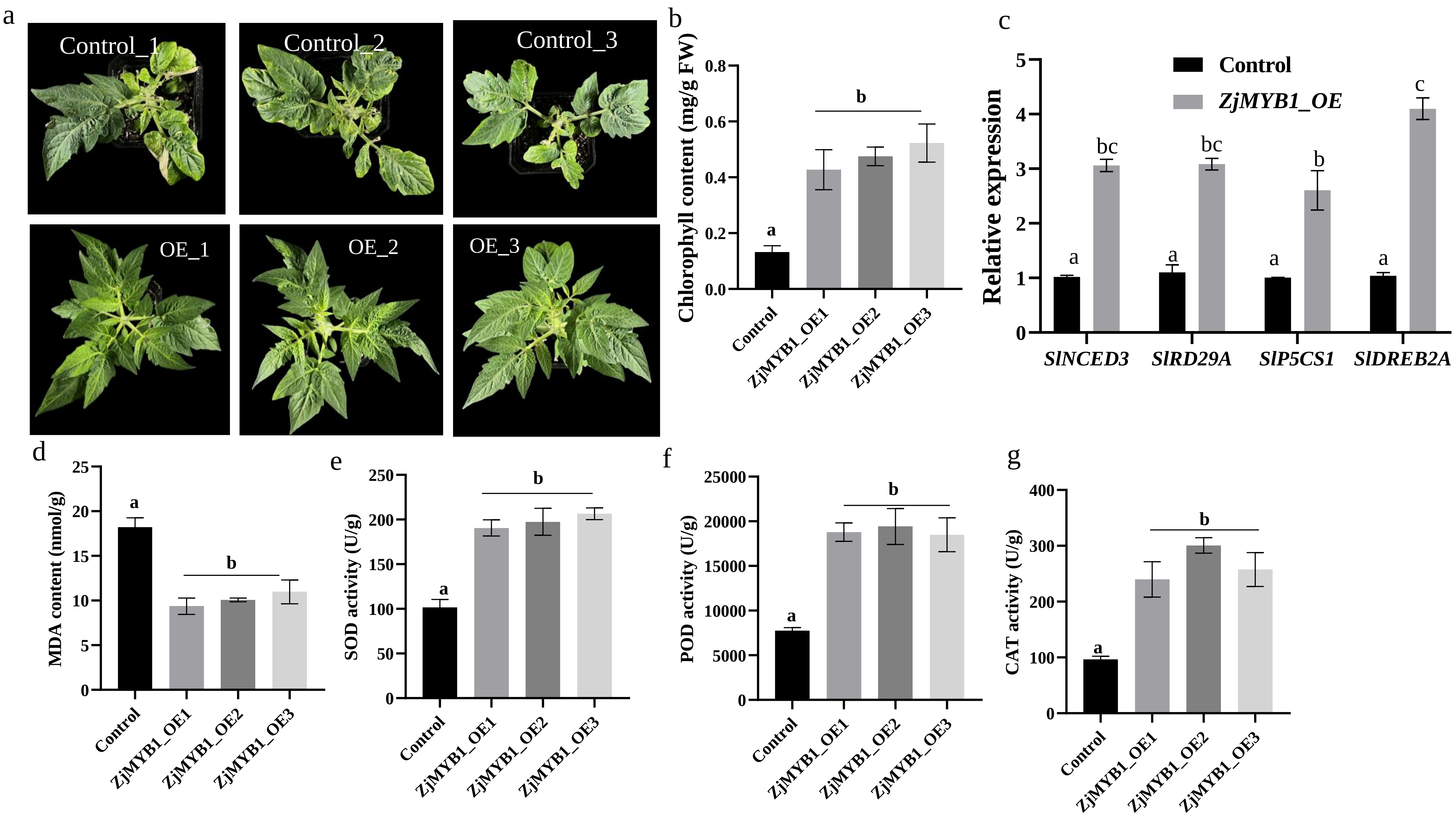

Figure S6. Effect of ZjMYB1 overexpression on defense-related genes and salt stress in tomato.(a) Growth phenotypes of overexpressed ZjMYB1 tomato and control under salt stress; (b) The difference of chlorophyll content between overexpressed and control lines; (c) Expression levels of defense related-genes; (d-g) The analysis of MDA content and SOD, POD, and CAT activities in overexpressed and control lines. Different letters (a–c) indicate significant differences according to one-way ANOVA ($p<0.05$). Error bars indicate mean ± SE.

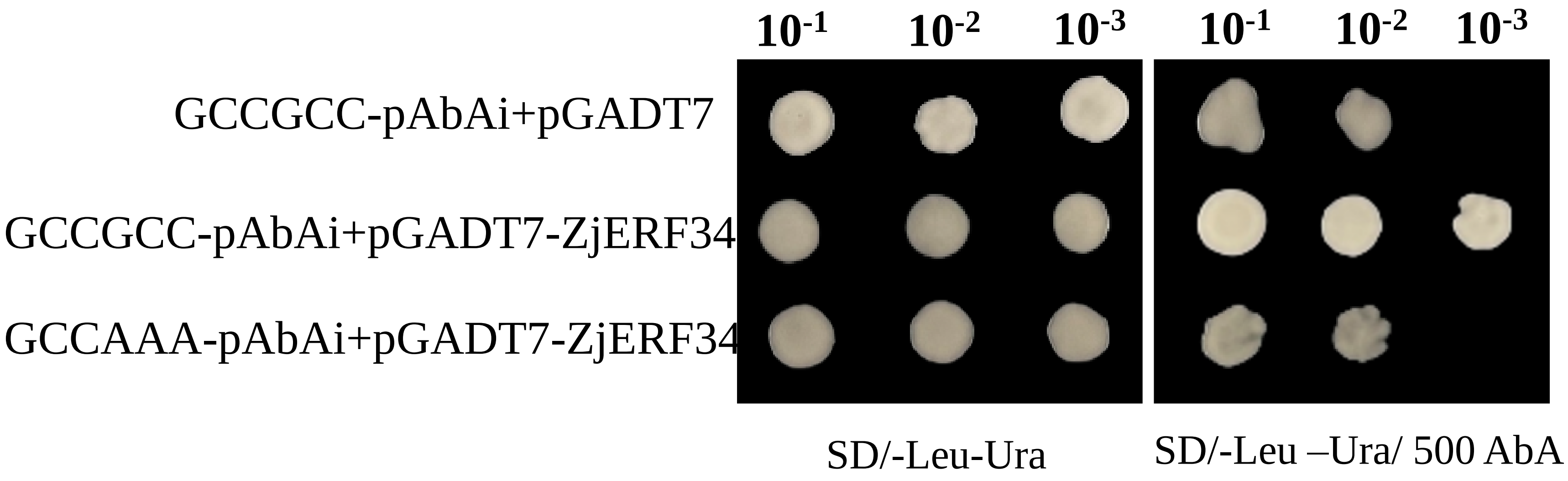

Figure S7. ZjERF34 specifically binds to the GCCGCC sequence of the ZjMYB1 promoter.
